# Autistic behavior is a common outcome of biallelic disruption of PDZD8 in humans and mice

**DOI:** 10.1186/s13229-025-00650-8

**Published:** 2025-02-27

**Authors:** Andreea D. Pantiru, Stijn Van de Sompele, Clemence Ligneul, Camille Chatelain, Christophe Barrea, Jason P. Lerch, Beatrice M. Filippi, Serpil Alkan, Elfride De Baere, Jamie Johnston, Steven J. Clapcote

**Affiliations:** 1 School of Biomedical Sciences, University of Leeds, Leeds, LS2 9JT UK; 2 Division of Neuroscience, School of Biological Sciences, University of Manchester, Manchester, M13 9PT UK; 3 Center for Medical Genetics, Ghent University Hospital, Ghent, Belgium; 4 Department of Biomolecular Medicine, Ghent University, Ghent, Belgium; 5 Wellcome Centre for Integrative Neuroimaging, Nuffield Department of Clinical Neuroscience, University of Oxford, Oxford, OX1 3SR UK; 6 Department of Human Genetics, University Hospital of Liege, Liege, Belgium; 7 Autism Resource Centre of Liege, University of Liege, Liege, Belgium

**Keywords:** Autism spectrum disorder, Intellectual disability, Olfactory behavior, PDZD8, Social discrimination

## Abstract

**Background:**

Intellectual developmental disorder with autism and dysmorphic facies (IDDADF) is a rare syndromic intellectual disability (ID) caused by homozygous disruption of PDZD8 (PDZ domain-containing protein 8), an integral endoplasmic reticulum (ER) protein. All four previously identified IDDADF cases exhibit autistic behavior, with autism spectrum disorder (ASD) diagnosed in three cases. To determine whether autistic behavior is a common outcome of PDZD8 disruption, we studied a third family with biallelic mutation of *PDZD8* (family C) and further characterized PDZD8-deficient (*Pdzd8*^*tm1b*^) mice that exhibit stereotyped motor behavior relevant to ASD.

**Methods:**

Homozygosity mapping, whole-exome sequencing, and cosegregation analysis were used to identify the *PDZD8* variant responsible for IDDADF, including diagnoses of ASD, in consanguineous family C. To assess the in vivo effect of PDZD8 disruption on social responses and related phenotypes, behavioral, structural magnetic resonance imaging, and microscopy analyses were conducted on the *Pdzd8*^*tm1b*^ mouse line. Metabolic activity was profiled using sealed metabolic cages.

**Results:**

The discovery of a third family with IDDADF caused by biallelic disruption of PDZD8 permitted identification of a core clinical phenotype consisting of developmental delay, ID, autism, and facial dysmorphism. In addition to impairments in social recognition and social odor discrimination, *Pdzd8*^*tm1b*^ mice exhibit increases in locomotor activity (dark phase only) and metabolic rate (both lights-on and dark phases), and decreased plasma triglyceride in males. In the brain, *Pdzd8*^*tm1b*^ mice exhibit increased levels of accessory olfactory bulb volume, primary olfactory cortex volume, dendritic spine density, and ER stress- and mitochondrial fusion-related transcripts, as well as decreased levels of cerebellar nuclei volume and adult neurogenesis.

**Limitations:**

The total number of known cases of *PDZD8*-related IDDADF remains low. Some mouse experiments in the study did not use balanced numbers of males and females. The assessment of ER stress and mitochondrial fusion markers did not extend beyond mRNA levels.

**Conclusions:**

Our finding that the *Pdzd8*^*tm1b*^ mouse model and all six known cases of IDDADF exhibit autistic behavior, with ASD diagnosed in five cases, identifies this trait as a common outcome of biallelic disruption of PDZD8 in humans and mice. Other abnormalities exhibited by *Pdzd8*^*tm1b*^ mice suggest that the range of comorbidities associated with PDZD8 deficiency may be wider than presently recognized.

**Supplementary Information:**

The online version contains supplementary material available at 10.1186/s13229-025-00650-8.

## Background

Intellectual disability (ID) is a genetically heterogeneous neurodevelopmental disorder affecting 1–3% of the general population [1]. Intellectual developmental disorder with autism and dysmorphic facies (IDDADF; OMIM #620021) is a very rare syndromic ID caused by homozygous premature termination codons (PTCs) in *PDZD8*, encoding PDZ domain-containing protein 8 (PDZD8) [2]. All four previously identified individuals with IDDADF, from two families, exhibit autistic behavior, with autism spectrum disorder (ASD) diagnosed in three individuals [2]. Two individuals with IDDADF also present with attention-deficit/hyperactivity disorder (ADHD) [2].

PDZD8 is an integral endoplasmic reticulum (ER) transmembrane protein that mediates the transfer of lipids from the ER to late endosomes and lysosomes, thereby promoting endosomal maturation and maintaining neuronal integrity [3-6]. PDZD8 also plays a role in regulating cytoplasmic Ca^2+^ dynamics in neurons following synaptic transmission-induced intracellular Ca^2+^ release from ER stores, by regulating mitochondrial uptake of Ca^2+^ [7-9]. Additionally, AMP-activated protein kinase (AMPK) activation-induced phosphorylation of PDZD8 at threonine 527 (pT527) is required for the increased utilization of glutamine (glutaminolysis) in response to hypoglycemia [10], and consequently for the extension of healthspan and lifespan induced by calorie restriction [11].

Given the limited number of patients and lack of natural history studies and post-mortem data, mouse models are instrumental in deciphering the pathophysiology and mechanisms underlying IDDADF. The *Pdzd8*^*tm1b*^ mouse model for IDDADF, which is homozygous for a frameshift and a PTC (p.F333Nfs1*), exhibits spontaneous repetitive hindlimb jumping [2], a stereotyped motor behavior relevant to lower-order human motor stereotypies that are common in ASD [1]. To determine whether autistic behavior is a common outcome of PDZD8 disruption, we identified a third family in which a homozygous mutation in *PDZD8* cosegregates with syndromic ID, and further profiled the PDZD8-deficient *Pdzd8*^*tm1b*^ mouse line.

Herein, we report that the *Pdzd8*^*tm1b*^ mouse model and all six known cases of IDDADF exhibit autistic behavior, with ASD diagnosed in five cases. In addition to impairments in social recognition and social odor discrimination, *Pdzd8*^*tm1b*^ mice with a C57BL/6NTac genetic background exhibit increases in locomotor activity (dark phase only) and metabolic rate (both lights-on and dark phases), and decreased plasma triglyceride in males. In the brain, *Pdzd8*^*tm1b*^ mice exhibit increased levels of accessory olfactory bulb volume, primary olfactory cortex volume, dendritic spine density, and ER stress- and mitochondrial fusion-related transcripts, as well as decreased levels of cerebellar nuclei volume and adult neurogenesis.

These findings suggest that PDZD8 deficiency may lead to atypical social responses and autistic behavior along with somatic features consistent with the syndromic nature of *PDZD8*-related ID.

## Methods

### Patients and clinical evaluation

**Fig. 1:**
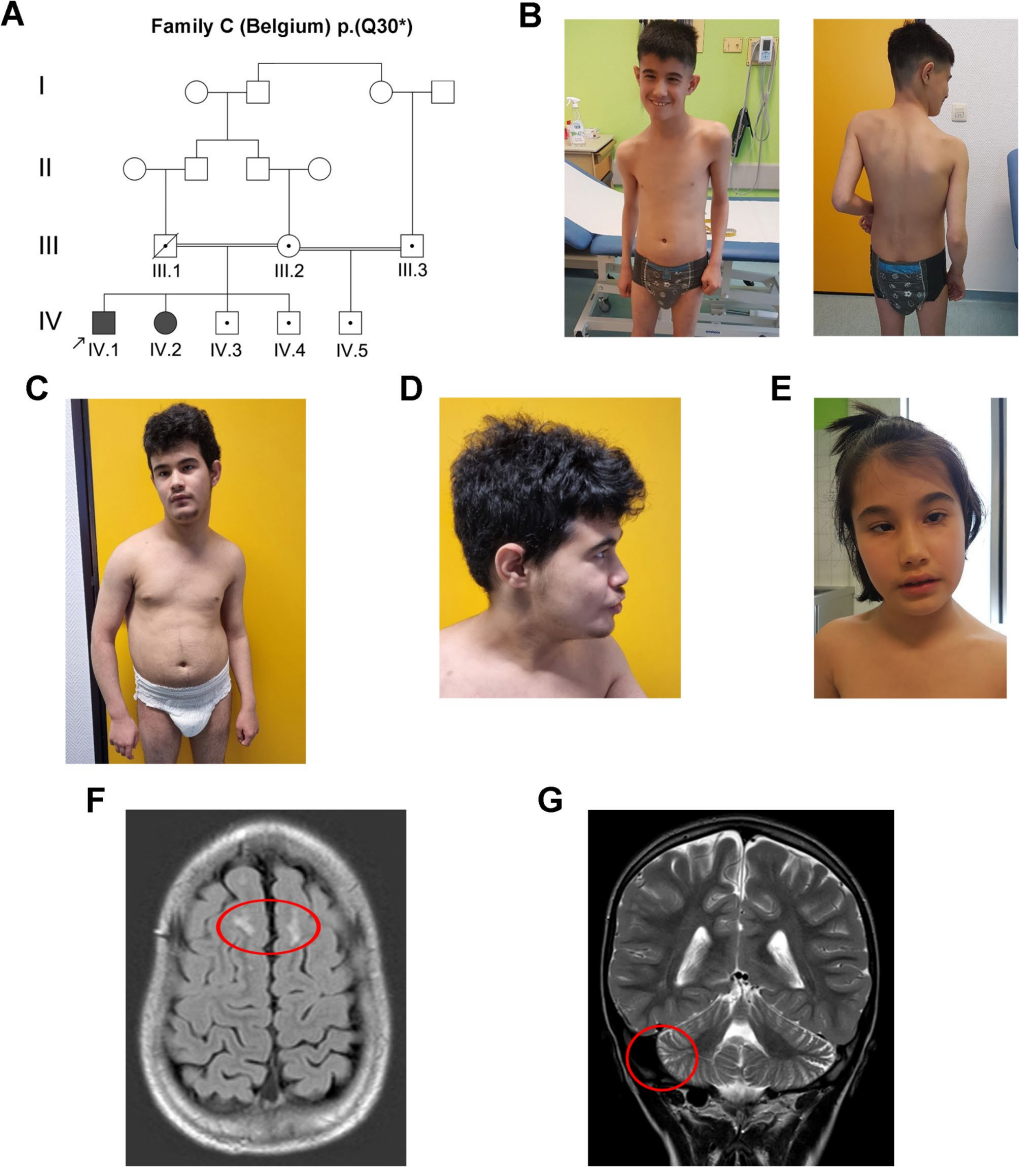
Clinical features of syndromic ID in family C. **A** Pedigree of four-generation family C showing cosegregation of *PDZD8* p.(Q30*) homozygosity with syndromic ID in 2 affected siblings. The obligate carrier status of the deceased father (C.III.1) of the affected siblings was not confirmed by genetic testing. Arrow, index case (C.IV.1); filled symbol, affected (symptomatic); open symbol, unaffected (asymptomatic); black dot, heterozygous carrier; diagonal line, deceased. **B** Scoliosis in index case (C.IV.1) aged 13 years. **C** Scoliosis in C.IV.1 aged 18 years. **D** Facial dysmorphism in C.IV.1 aged 18 years. **E** Facial dysmorphism including malar flattening in C.IV.2 aged 13 years. **F** Brain MRI scan showing demyelinating lesions in C.IV.1. **G** Brain MRI scan showing mild cerebellar hemispheric atrophy in C.IV.1

Family C was recruited through a clinic in the Department of Pediatrics at the University Hospital of Liege and consists of an affected 18-year-old male (C.IV.1), an affected 13-year-old female (C.IV.2), and two unaffected males aged 16 years (C.IV.3) and 11 years (C.IV.4). Their parents (C.III.1 and C.III.2) are first cousins within a pedigree of Afghan origin (Fig. 1A). The mother of these siblings has an unaffected 6-month-old child (C.IV.4) with a first cousin once removed (C.III.3).

Growth parameters were measured using the World Health Organization child growth standards [12], IQ was measured using the Stanford-Binet intelligence test [13], and adaptative behavior was assessed using the Vineland Adaptive Behavior Scales, 2nd edition (Vineland-II) [14] and the Psychoeducational Profile, 3rd edition (PEP-3) [15]. ASD was evaluated in accordance with DSM-5 autism diagnostic criteria [1] using the Childhood Autism Rating Scale, 2nd edition (CARS2) [16] (C.IV.1) and the Autism Diagnostic Observation Schedule, 2nd edition (ADOS-2) [17] (C.IV.2) (Additional file 1).

### Sequencing and variant identification

Peripheral blood was sampled by venipuncture for genomic DNA extraction using the MagCore Genomic DNA Large Volume Whole Blood Kit (RBC Bioscience, Freiburg, Germany). Whole-exome sequencing (WES) was conducted as described previously [18]. Briefly, exome enrichment was performed with the KAPA HyperExome Kit (Roche, Machelen, Belgium), followed by paired-end sequencing on a NovaSeq 6000 system (Illumina, Mechelen, Belgium). Sequencing reads were mapped against GRCh38/hg38 using BWA-MEM (version 0.7.17) and variant calling was performed using GATK HaplotypeCaller (version 3.8). To check for homozygous regions in the exome data, the AutoMap algorithm was used [19]. Variants were prioritized for further analysis based on presence in homozygous regions and CADD (Combined Annotation-Dependent Depletion) scores [20]. Copy number variant (CNV) analysis of the WES data, using ExomeDepth, did not detect any CNV affecting *PDZD8*. Segregation in the family was confirmed by polymerase chain reaction (PCR) and Sanger sequencing using the BigDye Terminator v3.1 kit (Applied Biosystems, Lennik, Belgium). Oligonucleotide primers were designed using Primer3 [21].

### Mice

C57BL/6NTac-*Pdzd8*^*tm1b(EUCOMM)Wtsi*^/WtsiH (PDZ domain containing 8; targeted mutation 1b, Wellcome Trust Sanger Institute) mice were obtained from the European Mouse Mutant Archive node at the National Mouse Archive, MRC Harwell, UK (www.infrafrontier.eu/emma/strain-search/straindetails/?q=14234) [22]. Briefly, the line was generated on a C57BL/6NTac genetic background through replacement of an 835-bp sequence including exon 3 by a *lacZ* expression cassette, which created a frameshift and a termination codon (p.F333Nfs1*) [2]. Heterozygotes were intercrossed to generate *Pdzd8*^*tm1b*^ homozygous mutant (*Pdzd8*^*tm1b*^; *tm1b*) and wild-type (WT) littermates for phenotypic testing. Pups were weaned at 4 weeks of age and grouped housed (3–5 mice/cage) with same-sex littermates under a 12-hour light/dark cycle (lights on at 06:00 and off at 18:00). Pelleted feed (CRM-P, SDS Diets, Braintree, UK) and water were provided *ad libitum*. DNA was extracted from ear biopsies taken at weaning. Mice were genotyped by multiplex PCR as described previously [2].

### Juvenile social interaction

Juvenile social interaction was assessed in female *Pdzd8*^*tm1b*^ mice and WT littermate controls (*n* = 8♀/genotype). After the experimental mouse was habituated to an empty cage for 5 min, a novel juvenile same-sex WT conspecific (C57BL/6J, 21 days old) was placed into the cage. The experimental mouse was allowed to freely interact with the novel juvenile mouse for 5 min. A different juvenile mouse was used for each experimental animal. All trials were recorded with ANY-maze Video Tracking Software (Stoelting, Dublin, Ireland). Video recordings were subsequently reviewed using Python Video Annotator (github.com/video-annotator/pythonvideoannotator) to identify periods of social interaction (categorized into approaches, anogenital sniffing, and other interactions). The total length of the social interaction periods was measured.

### Sociability and preference for social novelty

Sociability and preference for social novelty were assessed in female *Pdzd8*^*tm1b*^ mice (*n* = 12♀) and WT littermate controls (*n* = 10♀) using the three-chamber social approach test, as described previously [23]. After being habituated to an empty arena for 10 min, mice underwent two 10-minute trials. In the first trial, mice were exposed to a wire cylinder containing an unfamiliar female C57BL/6J mouse (aged 10 weeks; “stranger 1”) and a novel nonsocial/inanimate object (an empty wire cylinder). In the second trial, the previously empty cylinder had a second unfamiliar female mouse placed into it (“stranger 2”). All trials were video recorded and the time exploring stranger 1, the empty cylinder and stranger 2 was measured using ANY-maze software.

### Olfactory habituation and social discrimination

Olfactory habituation and social discrimination in *Pdzd8*^*tm1b*^ mice (*n* = 16; 8♂, 8♀) and WT littermate controls (*n* = 21; 13♂, 8♀) of both sexes were assessed in a 25 × 25 cm arena connected to an olfactometer (220 A, Aurora Scientific, Keynsham, UK) that delivered socially relevant odors, male and female urine (1:3 dilution in mineral oil; BioIVT, Burgess Hill, UK), and a non-socially relevant odor, isoamyl acetate (0.001% in mineral oil). The test consisted of a 10-minute habituation period, and four 1-minute presentations of each odor separated by 1-minute in the following order: air (1,000 SCCM), isoamyl acetate, female urine, and male urine. An Arduino controlled sensor, based on PROBES (poking registered olfactory behaviour evaluation system) [24], was used to measure the investigation time following each odor delivery.

### Olfactory detection test

*Pdzd8*^*tm1b*^ mice (*n* = 3; 2♂, 1♀) were anesthetized with isoflurane (~ 1.5–2%) on a custom stereotaxic frame for head-bar attachment. The skin above the skull was removed and cleaned with a sterile saline solution. Superglue was initially applied over the exposed skull followed by dental cement to affix a custom 3D printed head bar. Additional dental cement was applied to cover the head bar and the exposed skull. Mice were handled for 5 min each day for 2 days prior to behavioral testing. Mice were head-fixed on a treadmill, as described previously [25], and habituated for 10 to 20 min per day for 2 to 3 days before recordings. The mouse face was imaged with a PlayStation 3 Eye camera (Sony Computer Entertainment, Foster City, USA) with videos captured at 30 Hz. Odors were delivered using an olfactometer (Aurora Scientific) and custom-written code. A rectangular region of interest around the nose of the mouse was manually drawn and the frame-to-frame difference was extracted to measure nasal movements. Fourier analysis of this signal reveals oscillations around the respiratory rate of mice and odor evoked increases in the active sniffing range [26].

### Metabolic activity assessment

Adult male *Pdzd8*^*tm1b*^ mice and WT littermate controls (*n* = 9♂/genotype) were individually housed in sealed Comprehensive Lab Animal Monitoring System (CLAMS, Columbus Instruments, Ohio, USA) Perspex metabolic cages for 7 days and maintained under a 12-hour light/dark cycle (lights on at 06:00 and off at 18:00) at constant temperature (∼18 °C) and humidity (∼40%). The metabolic cages were connected through an open-circuit gas flow system provided with a known concentration of O_2_ and CO_2_ to allow constant, indirect calorimetric assessment. Pelleted feed (CRM-P, SDS Diets) was provided in an open access food hopper. Water was provided *ad libitum* through a plastic water bottle in the roof of the cage with a metallic sipper. All activity was recorded in 14-minute bins for analysis.

### Plasma triglyceride level measurement

Plasma triglyceride level measurements (mg/dL), freely available from the International Mouse Phenotyping Consortium (IMPC) portal (www.mousephenotype.org) [27], in retro-orbital blood samples from anesthetized 16-week-old *Pdzd8*^*tm1b*^ mice (*n* = 14; 7♂, 7♀) and C57BL/6NTac background strain controls (*n* = 280; 132♂, 148♀) of both sexes were obtained using an AU680 clinical chemistry analyzer (Beckman Coulter, Brea, USA) at MRC Harwell, in accordance with the IMPReSS (International Mouse Phenotyping Resource of Standardised Screens) clinical chemistry phenotyping protocol.

### Structural magnetic resonance imaging

To assess high-resolution structural magnetic resonance imaging (MRI) data for genotypic differences in specific brain regions, we normalized the volume of each region to the overall brain volume, using the formula [individual absolute volume region / individual absolute volume whole brain * mean absolute volume whole brain], and reported the normalized volume as % total brain volume. A linear model with genotype and sex as predictors was fitted to the absolute (mm^3^) and relative volume of every region independently and to every voxel independently in the brains of *Pdzd8*^*tm1b*^ mice (*n* = 32; 10♂, 22♀) and WT littermate controls (*n* = 17; 7♂, 10♀) of both sexes, with a false discovery rate (FDR) threshold of 5%. Multiple comparisons were controlled for using the FDR within the RMINC package for R, as described previously [2].

### Neurogenesis

EdU (5-ethynyl-2′-deoxyuridine) staining was performed to assess neurogenesis. Briefly, mice were given one dose of EdU (50 mg/kg, intraperitoneal), then perfused with 0.1 M phosphate-buffered saline (PBS), and their brains were fixed in 4% paraformaldehyde for 7 days. Sequential coronal sections of the OB and hippocampus in the right hemisphere were taken from *Pdzd8*^*tm1b*^ mice and WT littermate controls (*n* > 50 sections from *n* = 5♀/genotype). EdU reaction was performed in the presence of 2 M Tris, 5 mM CuSO_4_, 1 mM biotinylated azide and 0.5 M ascorbic acid, before final incubation with Alexa Fluor 555 streptavidin (1:1,000; Invitrogen, Paisley, UK). Sections were visualized using an AxioScan Slide Scanner (Carl Zeiss, Cambourne, UK) at ×20 magnification. Image analysis was performed in Cellpose [28], an anatomical segmentation algorithm written in Python 3, using the nucleus model. The total number of EdU puncta per section was calculated and normalized by surface area. The OB was divided into granule cell layer and extra granule cell layer, while the hippocampus was examined as a whole due to the lower number of EdU cells detected.

### Dendritic spine analysis

A Golgi–Cox impregnation kit (FD Rapid GolgiStain Kit; FD NeuroTechnologies, Columbia, USA) was used for dendritic spine count analysis. Briefly, *Pdzd8*^*tm1b*^ mice and WT littermate controls (*n* = 4♂/genotype) were anesthetized with isoflurane and then decapitated. Brains were extracted and the right brain hemisphere was immersed in 4 ml impregnation solution for 7 days. The brain tissue was then placed in Solution C for 3 days. Sequential coronal sections of the right hemisphere (100 μm) were washed in 0.1 M PBS, placed in developing solution for 10 min, and then washed 3 times in 0.1 M PBS. Sections were mounted on gelatine-coated slides and dehydrated through a series of ethanol washes (50%, 75%, 95%, 100%; 4 min each). Sections were cleared using Histo-Clear (National Diagnostics, Atlanta, USA) for 10 min and then visualized using an AxioScan Slide Scanner (Carl Zeiss) at ×20 magnification. In OB and hippocampus (CA1 and dentate gyrus), dendritic spines on secondary and tertiary dendrites (at least two dendrites from different neurons in each brain section) were counted over a 10-µm length (∼16–25 dendrites/mouse) using ZEN Microscopy Software (Carl Zeiss).

### Transcriptional analysis

Cervical dislocation was performed and mouse brains were extracted and snap frozen in liquid nitrogen. Messenger RNA (mRNA) was extracted using an RNAqueous Total RNA Isolation Kit (Invitrogen) from whole brains of male *Pdzd8*^*tm1b*^ mice and WT littermate controls (*n* = 5♂/genotype). A Nanodrop 2000 spectrophotometer (Thermo Scientific, Altrincham, UK) was used to measure RNA concentration (260 nm) and purity (260/280 nm ratio). Subsequently, cDNA was synthesized using SuperScript III Reverse Transcriptase (Invitrogen) from 1 µg of mRNA per sample and stored at − 80 °C before analysis by qRT-PCR. Using PowerTrack SYBR Green Master Mix (Applied Biosystems, Warrington, UK), the cDNA was amplified using the following program in a QuantStudio 3 Real-Time PCR System (Applied Biosystems): 10 min at 95 °C, 15 s at 95 °C and 1 min at 60 °C repeated 40 times, then 5 s at 60 °C. The following oligonucleotide primers designed using Primer-BLAST [29] were utilized: *Atf4*: forward (5′-CAG ACA CCG GCA AGG AGG AT-3′) and reverse (5′-AAG AGC TCA TCT GGC ATG GT-3′); *B2m*: forward (5′-CTG GTG CTT GTC TCA CTG ACC-3′) and reverse (5′-CGT AGC AGT TCA GTA TGT TCG G-3′); *Fis1*: forward (5′-CTG TGG AGG ATC TGA AGA ATT TTG-3′) and reverse (5′-AAC CAG GCA CCA GGC ATA TT-3′); *Hprt*: forward (5′-TGC TGA CCT GCT GGA TTA CAT-3′) and reverse (5′-TTT ATG TCC CCC GTT GAC TGA T-3′); *Hspa5*: forward (5′-CGT GTG TGT GAG ACC AGA AC-3′) and reverse (5′-GCC ACC ACA GTG AAC TTC ATC A-3′); *Mfn1*: forward (5′-CAG AAA GCA TAA AGC TCA GGG G-3′) and reverse (5′-GAC TGC GAG ATA CAC TCC TCA A-3′); *Mfn2*: forward (5′-CCA GCT AGA AAC TTC TCC TCT GTT-3′) and reverse (5′-AGG GAC ATC TCG CCA GTT TA-3′); *Opa1*: forward (5′-TGA GGC CCT TCT CTT GTT AGG T-3′) and reverse (5′-CTT TTC TTT GTC TGA CAC CTT CCT-3′). Data were normalized to the *Hprt* and *B2m* reference genes. Analysis was carried out using the 2^-ΔΔCt^ method [30].

### Statistical analysis

Statistical analysis was performed using GraphPad Prism or SciPy and Pingouin libraries in Python. Data were assessed for normality using the Shapiro–Wilk test. Data passing normality assumptions were analyzed using Student’s *t*-test or two-way analysis of variance (ANOVA) with repeated measures, as necessary, followed by Tukey’s post hoc tests with statistical significance set at *p* < 0.05. If the data violated normality, Mann–Whitney tests were used. Experimenters were blinded to genotype during behavioral testing.

## Results

### *PDZD8* mutation in a third family with syndromic ID

Family C consists of two affected (C.IV.1 and C.IV.2) and two unaffected (C.IV.3 and C.IV.4) siblings born to consanguineous parents (first cousins) (C.III.1 and C.III.2) within a pedigree of Afghan origin (Fig. 1A). Neuropsychological assessments revealed that both the index case (C.IV.1) and his affected sister (C.IV.2) have severe ASD, severe ID, and a lack of functional language (Additional file 1). They present facial dysmorphism (hypotonic face, malar flattening, thin palpebral fissures, and open mouth), a proportional short stature, with a height more than three standard deviations below the mean for age and sex [12], no signs of skeletal dysplasia on radiographies, and mild myasthenia with a limited walking perimeter. In addition, C.IV.1 presents scoliosis and generalized epilepsy treated by valproic acid (VPA) and levetiracetam (Fig. 1B–E). Brain MRI revealed cortico-subcortical frontal and parietal demyelinating lesions with mild cerebellar hemispheric atrophy in C.IV.1 (Fig. 1F, G) and subcortical aspecific gliotic lesions in C.IV.2. The siblings’ father (C.III.1) is deceased; their mother (C.III.2) has an asymptomatic son (C.IV.5) with another consanguineous partner, a first cousin once removed (C.III.3). All heterozygous children and parents are asymptomatic.

**Fig. 2:**
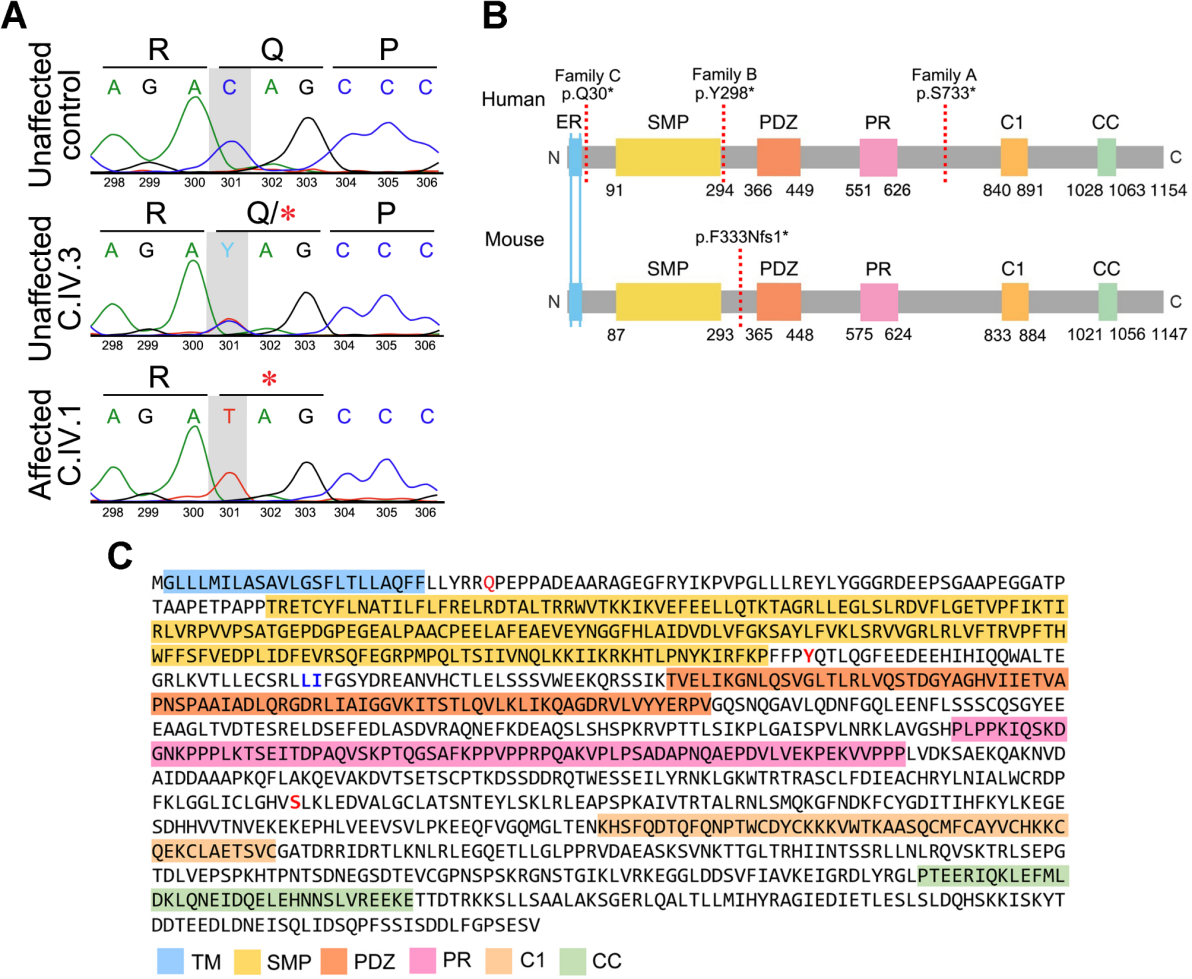
Overview of molecular findings in *PDZD8*-related IDDADF. **A** Sanger sequence chromatograms showing the *PDZD8* nonsense mutation (c.88 C > T) identified in family C. **B** Schematic diagram depicting domain structure of PDZD8 in human (UniProtKB: Q8NEN9; top) and mouse (UniProtKB: B9EJ80; bottom). Broken vertical red lines indicate the location of PTC in family A (p.S733*), family B (p.Y298*) and family C (p.Q30*), and in *Pdzd8*^*tm1b*^ mice (p.F333Nfs1*). Numbering is from reference [2]. **C** Location of the p.(Q30*), p.(Y298*) and p.(S733*) variants (red text) within protein sequence and domain organization of human PDZD8 (Q8NEN9). Blue text indicates the residues (L334 & I335) corresponding to F333 and I334 affected by p.(F333Nfs1*) in mouse PDZD8. C, carboxyl-terminus; C1, phorbol-ester/diacylglycerol-binding; CC, coiled-coil; ER, endoplasmic reticulum transmembrane; N, amino-terminus; PR, proline-rich; PDZ, PSD-95/DlgA/ZO-1-like; SMP, synaptotagmin-like mitochondrial lipid-binding

The pedigree structure of family C suggested autosomal recessive transmission of a homozygous mutant allele from a shared ancestor as the most likely explanation for the condition of the two affected siblings. Homozygosity mapping using variants extracted from WES of C.IV.1, with filtering for predicted pathogenic variants and segregation analysis, identified a homozygous nonsense variant in *PDZD8* exon 1 [GRCh38: chr10-117375140-G-A; NM_173791.5: c.88 C > T; p.(Q30*)] as likely causal (Fig. 2A). The p.(Q30*) variant has a CADD score of 35.0 and is present at a frequency of 6.287908226 × 10^− 7^ with no homozygotes in gnomAD version 4.1.0 [31]. If the p.(Q30*) mRNA transcript evades nonsense-mediated decay, it may be translated into a truncated, non-functional PDZD8 protein lacking 1,125 C-terminal amino acids (97.5%) from the 1,154-aa full-length protein (Fig. 2B, C).

As the affected individuals in family C exhibit a similar clinical profile and molecular diagnosis to the previously described cases with homozygous PTCs in *PDZD8* (Table 1), they constitute the fifth and sixth individuals and the third family diagnosed with IDDADF resulting from mutation of *PDZD8*. Examination of the clinical features shared by all six known cases of IDDADF revealed a core clinical phenotype of developmental delay, ID, autism, and facial dysmorphism (Table 1).

**Table 1:** Clinical features of patients with homozygous PTCs in *PDZD8*

Characteristic	Family C Affected Individuals		Incidence in all IDDADF cases [2]
	C.IV.1	C.IV.2	
Consanguinity	Yes	Yes	6/6
Ethnic Origin	Afghan	Afghan	
Genotype, Mat/Pat	p.(Q30*)/p.(Q30*); c.88 C > T/c.88 C > T	p.(Q30*)/p.(Q30*); c.88 C > T/c.88 C > T	
Sex	Male	Female	
Age, Years	18	13	
Developmental Delay	Yes	Yes	6/6
Intellectual Disability	Yes (severe)	Yes (severe)	6/6 (severe: 4/6)
Autism	Yes (ASD)	Yes (ASD)	6/6 (ASD: 5/6)
Facial Dysmorphism	Yes	Yes	6/6
Myasthenia	Yes (mild)	Yes (mild)	5/6 (mild: 3/6)
Epilepsy	Yes	No	3/6
Scoliosis	Yes	No	2/6
Aggression	No	Yes	1/6
Orbital Hypertelorism	No	No	4/6
Myopia	No	No	2/6
Marfanoid Habitus	No	No	2/6
ADHD	No	No	2/6
Brain Scan Findings	Cortico-subcortical demyelinating lesions; mild cerebellar atrophy	Subcortical aspecific gliotic lesions	

Nucleotide and residue numbering are based on NM_173791.5

ADHD, attention-deficit/hyperactivity disorder; ASD, autism spectrum disorder; IDDADF, intellectual developmental disorder with autism and dysmorphic facies; Mat, maternal; Pat, paternal

Analysis of cross-species brain RNA-seq expression data from the Human Protein Atlas [32] revealed that PDZD8 is expressed throughout the mammalian brain, with low regional specificity (Additional file 2), consistent with the clinical phenotype of IDDADF and with published observations in WT mice [33]. Analysis of murine brain single-cell RNA-seq data from the Allen Cell Types Database [34] identified PDZD8 transcripts in nearly all subclasses of GABAergic inhibitory neurons and glutamatergic excitatory neurons and in oligodendrocytes (Additional file 3).

### Enhanced locomotor activity and metabolic rate in *Pdzd8*^*tm1b*^ mice

**Fig. 3:**
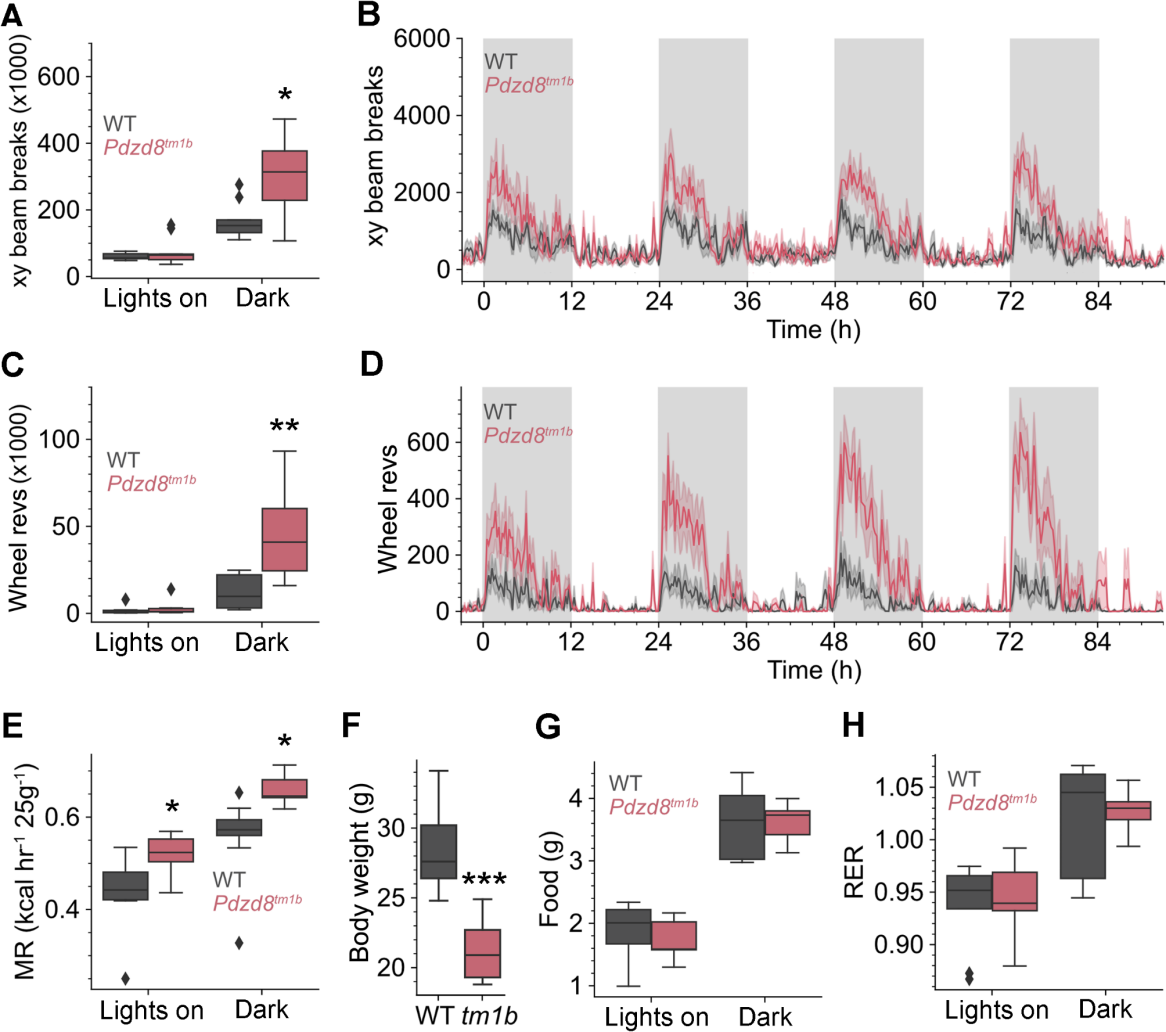
Enhanced locomotor activity and metabolic rate in *Pdzd8*^*tm1b*^ mice. **A** Locomotor activity on the cage floor (*x*–*y* beam breaks) was significantly different between *Pdzd8*^*tm1b*^ mice (*n* = 9♂) and WT controls (*n* = 9♂) (two-way ANOVA: *F* = 8.292, *p* = 0.00705). Post-hoc pairwise corrected *t*-tests showed a significant difference only during the dark phase (*t* = 2.654, *p* = 0.035). **B** Locomotor activity on the cage floor (*x*–*y* beam breaks) throughout the 12-hour light/dark cycle over 96 h. **C** Locomotor activity (in-cage running wheel revolutions) was significantly different between *Pdzd8*^*tm1b*^ mice and WT controls (two-way ANOVA: genotype: *F* = 12.441, *p* = 0.001294). Post-hoc pairwise corrected *t*-tests showed a significant difference only during the dark phase (*t* = 3.451, *p* = 0.0066). **D** Locomotor activity (in-cage running wheel revolutions) throughout the 12-hour light/dark cycle over 96 h. **E** Metabolic rate measured as mean hourly heat production was elevated in *Pdzd8*^*tm1b*^ mice (two-way ANCOVA: genotype: *F* = 4.783, *p* = 0.036). For an equivalent 25 g mouse, the metabolic rate was significantly elevated during both the lights-on phase (post-hoc pairwise corrected *t*-test: *t* = 2.461, *p* = 0.026) and dark phase (post-hoc pairwise corrected *t*-test: *t* = 3.073, *p* = 0.0073). **F** Reduced body weight (g) of male *Pdzd8*^*tm1b*^ mice versus WT controls at 11 weeks of age (unpaired *t*-test: *t* = 5.8603, *p* = 0.000026). **G** Unaltered food intake (g) in *Pdzd8*^*tm1b*^ mice versus WT controls. **H** Unaltered respiratory exchange ratio in *Pdzd8*^*tm1b*^ mice versus WT controls (two-way ANOVA: genotype: *F* = 0.269, *p* = 0.607). g, grams; MR, metabolic rate; RER, respiratory exchange ratio; revs, revolutions; *tm1b*, *Pdzd8*^*tm1b*^ homozygous; WT, wild-type. *p < 0.05, **p < 0.01, ***p < 0.001 versus WT

Given the short stature of the affected siblings in family C, the reduced body length and soft tissue mass of *Pdzd8*^*tm1b*^ mice [2], and the role of PDZD8 in cell metabolism [10, 11], we examined a cohort of male mice (*n* = 9♂/genotype) in metabolic cages under a 12-hour light/dark cycle. A free-spinning running wheel was placed in each metabolic cage because we had previously observed increased voluntary wheel running in a murine model for comorbid autism [35-37]. During the dark phase, *Pdzd8*^*tm1b*^ mice displayed significantly increased locomotor activity compared with WT littermate controls both on the cage floor (ambulation) (Fig. 3A, B) and on the running wheel (Fig. 3C, D). However, during the lights-on phase – the natural resting time of nocturnal mice [38] – locomotor activity was not significantly different between genotypes. By contrast, the metabolic rate of *Pdzd8*^*tm1b*^ mice was significantly higher than that of WT controls across both the dark and lights-on phases (Fig. 3E). The *Pdzd8*^*tm1b*^ males had significantly lower body weights (Fig. 3F), consistent with our published observations in a separate colony [2], yet they consumed the same amount of food as heavier WT controls (Fig. 3G). The respiratory exchange ratio (RER) of O_2_ consumption to CO_2_ production was unaltered in *Pdzd8*^*tm1b*^ mice, with the mean RER across the 12-hour light/dark cycle being ∼0.98 for both genotypes (Fig. 3H), indicative of carbohydrate as the predominant energy substrate [39]. Metabolic profiling of male *Pdzd8*^*tm1b*^ mice thus revealed a phenotype of increased in-cage locomotor activity and wheel running during the dark phase, and a stable increase in metabolic rate across the light/dark cycle.

**Fig. 4:**
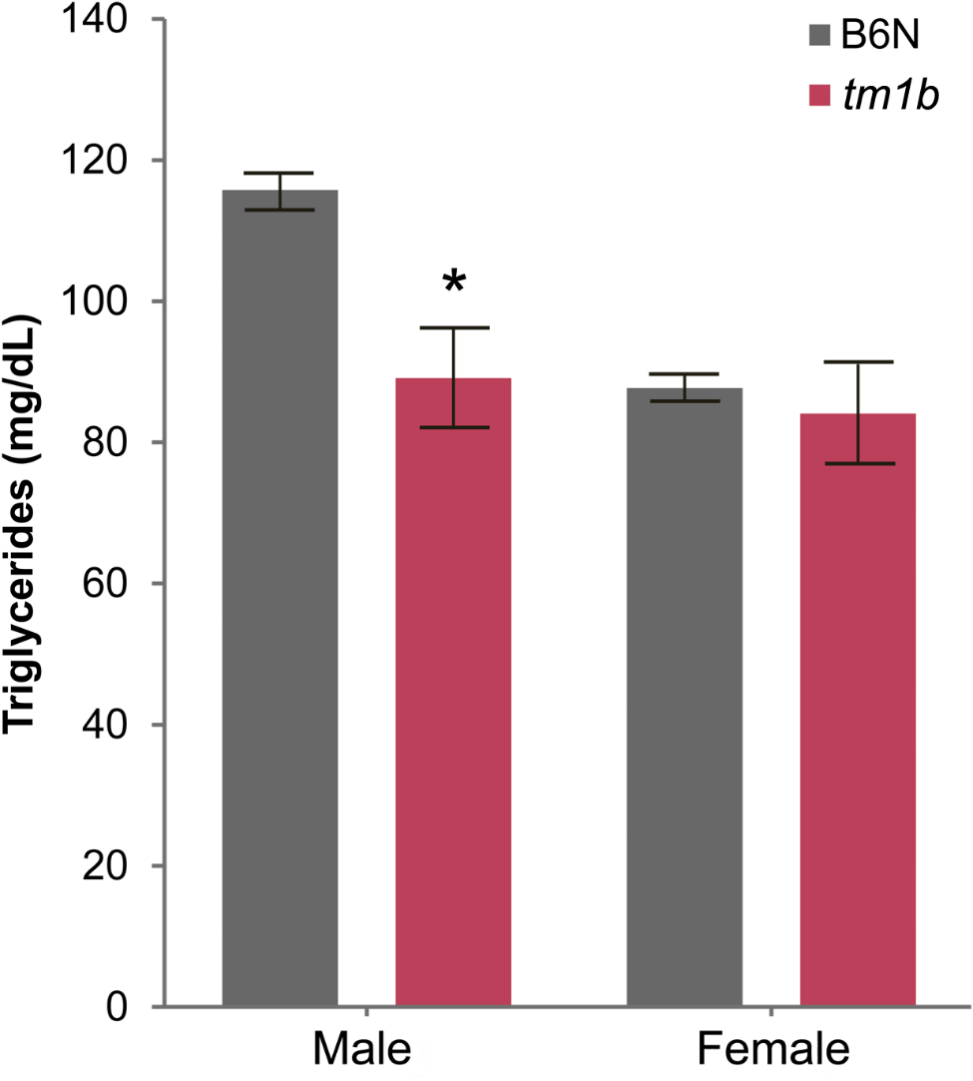
Decreased plasma triglyceride levels in male *Pdzd8*^*tm1b*^ mice. Plasma triglyceride levels (mg/dL) in *Pdzd8*^*tm1b*^ mice (*n* = 14; 7♂, 7♀) and C57BL/6NTac background strain controls (*n* = 280; 132♂, 148♀) (two-way ANOVA, genotype: *F*(1, 290) = 4.58, *p* = 0.033; sex: *F*(1, 290) = 5.50, *p* = 0.020; genotype × sex interaction: *F*(1, 290) = 2.65, *p* = 0.104). *p < 0.05 versus B6N ♂

Since physical exercise enhances energy expenditure and decreases plasma triglyceride levels in mice [40], we analyzed blood biochemistry data for the *Pdzd8*^*tm1b*^ mouse line from the IMPC portal [27]. This analysis revealed that plasma triglyceride is decreased by 22.99 ± 6.1% in male *Pdzd8*^*tm1b*^ mice (*n* = 7♂) compared with C57BL/6NTac background strain controls (*n* = 132♂) (post hoc Tukey: *t* = 2.66, *p* = 0.039), but among females the genotypes did not significantly differ (post hoc Tukey: *t* = 0.36, *p* = 0.984) (Fig. 4).

### Impaired social recognition and social odor discrimination in *Pdzd8*^*tm1b*^ mice

**Fig. 5:**
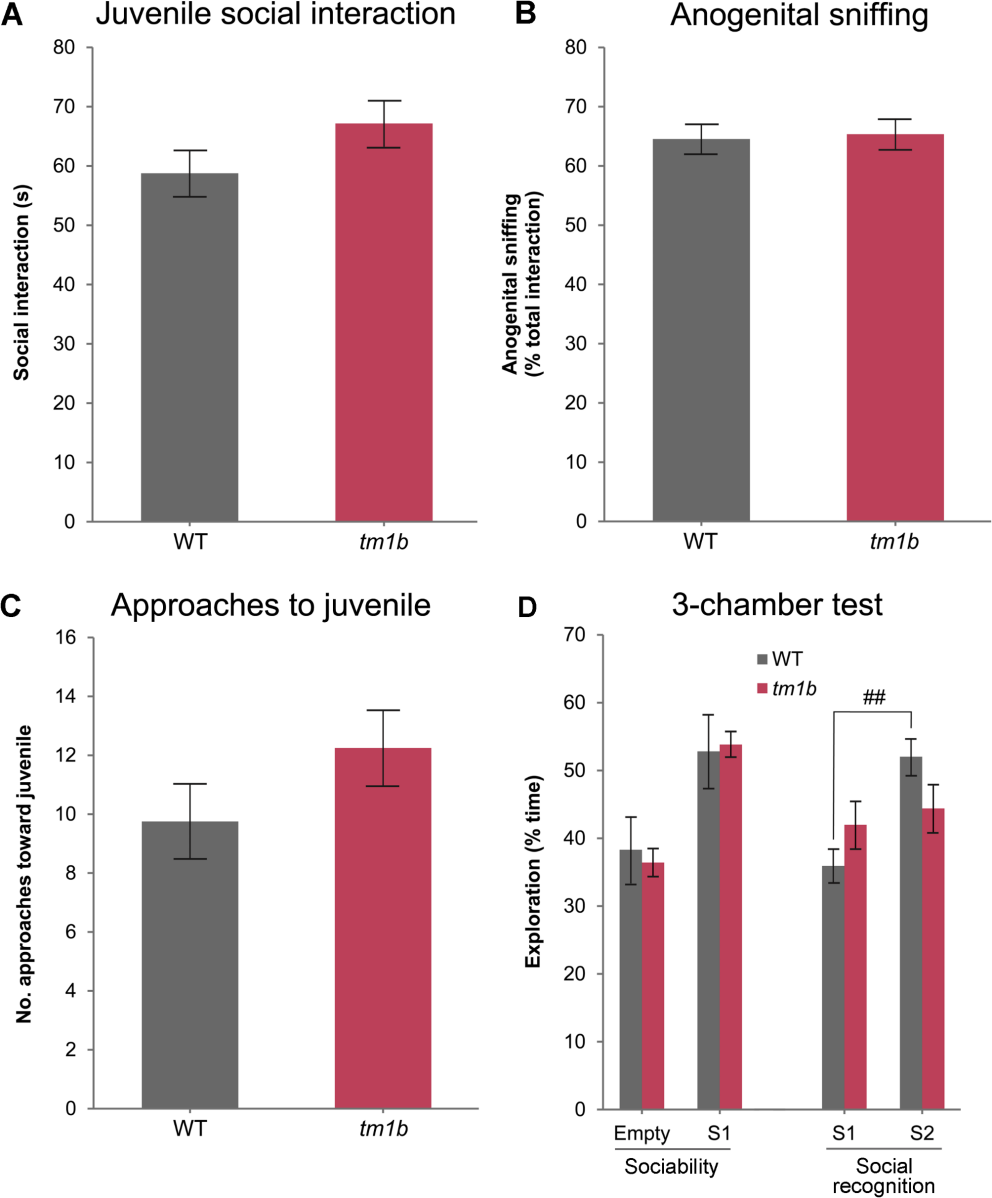
Impaired social recognition in *Pdzd8*^*tm1b*^ mice. **A–C** Juvenile social interaction in *Pdzd8*^*tm1b*^ mice (*n* = 8♀) and WT controls (*n* = 8♀). **A** Duration of social interaction (s). **B** Duration of anogenital sniffing (% of total interaction time). **C** Number of approaches toward juvenile mouse. **D** Social approach testing in *Pdzd8*^*tm1b*^ mice (*n* = 12♀) and WT controls (*n* = 10♀). Sociability: time (% total) spent exploring an empty container versus a novel mouse (two-way ANOVA, genotype: *F*(1, 40) = 0.01, *p* = 0.903; chamber: *F*(1, 40) = 19.16, *p* = 0.0001; genotype × chamber interaction: *F*(1, 40) = 0.16, *p* = 0.693). Social recognition: time (% total) spent exploring stranger 1 (previously explored mouse) versus a second novel mouse (two-way ANOVA, genotype: *F*(1, 40) = 0.06, *p* = 0.809; chamber: *F*(1, 40) = 8.50, *p* = 0.006; genotype × chamber interaction: *F*(1, 40) = 4.65, *p* = 0.037). Empty, empty cylinder; S1, stranger 1; S2, stranger 2; *tm1b*, *Pdzd8*^*tm1b*^ homozygous; WT, wild-type. ^##^*p* < 0.01 versus stranger 1 within WT group

Most autistic individuals show reduced or unusual social approach [41], while 15–30% exhibit severe deficits in face recognition, an integral part of human social interaction [42, 43]. In light of our finding that autism is a component of the core clinical phenotype of IDDADF, we examined the social behavior of female *Pdzd8*^*tm1b*^ mice and WT littermate controls. In a reciprocal social interaction test, within a neutral environment to which they had been habituated, both genotypes (*n* = 8♀/genotype) spent comparable amounts of time socially interacting with a freely moving female juvenile mouse over 5 min (Fig. 5A), including anogenital sniffing (Fig. 5B) and approaches toward the juvenile (Fig. 5C).

Similarly, in a three-chamber social approach test, *Pdzd8*^*tm1b*^ mice (*n* = 12♀) and WT littermate controls (*n* = 10♀) demonstrated comparable levels of sociability by spending more time (> 50%) in proximity to a novel mouse enclosed in a wire cylinder (stranger 1) versus a novel nonsocial/inanimate object, an empty wire cylinder (Fig. 5D). When subjects were subsequently given a choice between the first mouse (stranger 1) and a new mouse introduced into the previously empty cylinder (stranger 2), WT mice demonstrated a preference for social novelty by investigating stranger 2 more than the now familiar stranger 1 (post hoc Tukey, S1 versus S2: *t* = 3.43, *p* = 0.007). However, no such preference was shown by *Pdzd8*^*tm1b*^ mice (post hoc Tukey, S1 versus S2: *t* = 0.56, *p* = 0.943) (Fig. 5D), indicative of a deficit in social recognition.

**Fig. 6:**
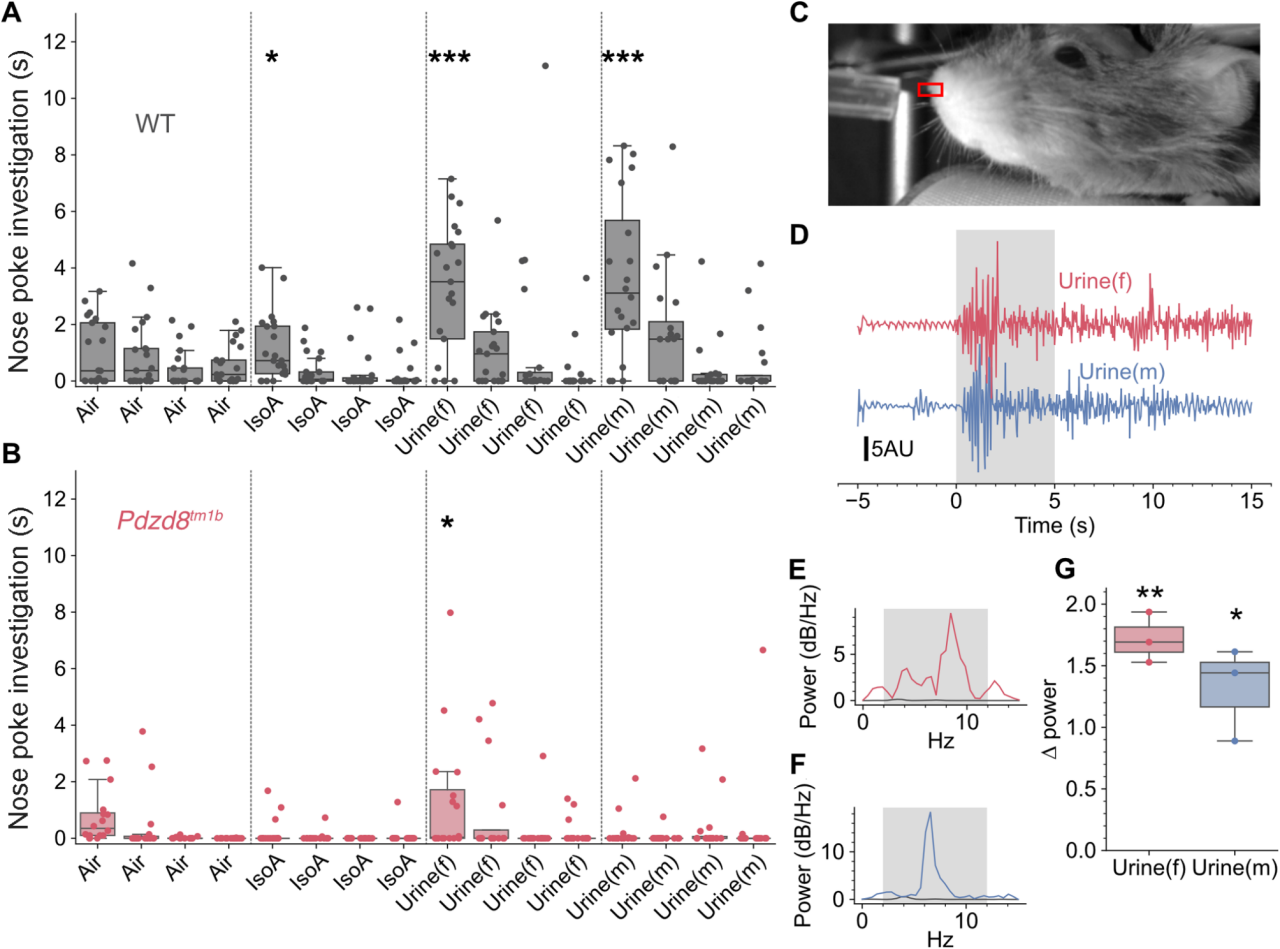
Social odour discrimination is altered in *Pdzd8*^*tm1b*^ mice. **A** Cross-habituation assay for WT controls (*n* = 21; 13♂, 8♀) showing increased nose poke investigation times (s) when a new odor was presented after habituation (Friedman: *F* = 3.389, *p* = 0.000007). **B** Cross-habituation assay for *Pdzd8*^*tm1b*^ mice (*n* = 16; 8♂, 8♀) showing investigation times lower than in WT controls but still significant (Friedman: *F* = 2.259, *p* = 0.0059). Asterisks indicate corrected post-hoc Wilcoxon tests. **C** A head-fixed *Pdzd8*^*tm1b*^ mouse, the rectangle over the nose showing the region used for analysis. **D** Nasal movements measured from the rectangle in B for female and male urine, with stimuli delivered during shaded area. **E**, **F** Fourier transforms of the data in D, the gray traces showing the power for the pre-stimulus and the colored traces showing the power over the stimulus period. Shaded area shows frequency range of respiration. **G***Pdzd8*^*tm1b*^ mice (*n* = 3; 2♂, 1♀) increased nasal movements in response to both female (corrected paired *t*-test: *t* = 14.507, *p* = 0.0094) and male urine (corrected paired *t*-test: *t* = 6.019, *p* = 0.027). dB, decibels; Hz, Hertz; IsoA, isoamyl acetate; Urine(f), urine from female mice; Urine(m), urine from male mice; WT, wild-type. *p < 0.05, **p < 0.01, ***p < 0.001

Olfaction is thought to play an important role in social recognition in rodents, enabling identification of conspecifics by their olfactory signature [44, 45]. As a deficiency in social odor discrimination has been displayed by the BTBR murine model for idiopathic autism [46], we investigated whether there is any deficit in the ability of *Pdzd8*^*tm1b*^ mice of both sexes to distinguish between socially relevant olfactory signatures. To do this, we performed an automated cross-habituation assay [25], in which nose poke investigation of an odor port indicates interest in the stimulus, and repeated presentations result in habituation (Fig. 6A, B). The ability to discriminate between pairs of odors can then be detected by increased investigation upon presentation of the new odor.

WT controls (*n* = 21; 13♂, 8♀) displayed significant differences in nose poke investigation times to the different stimuli (Fig. 6A). After habituating to the carrier air stream, WT controls investigated the neutral odor isoamyl acetate (post-hoc corrected Wilcoxon: *Z* = 2.163, *p* = 0.03) and then mouse urine, a socially relevant odor. Importantly, WT mice could discriminate between female and male urine (post-hoc corrected Wilcoxon: *Z* = 4.325, *p* = 0.00076). *Pdzd8*^*tm1b*^ mice (*n* = 16; 8♂, 8♀) had lower overall investigation times than WT controls (Mann–Whitney: *U* = 281.0, *p* = 0.00056) but did show differences in nose poke investigation times across stimuli (Fig. 6B). *Pdzd8*^*tm1b*^ mice investigated when urine was initially presented (post-hoc corrected Wilcoxon: *Z* = 2.335, *p* = 0.049) but, unlike WT controls, they failed to investigate when the urine was switched from female to male (post-hoc corrected Wilcoxon: *Z* = -7.68 × 10^–17^, *p* = 1.0), implying that they are unable to discriminate between these socially relevant odors. There was no sex effect, as males and females within each genotype exhibited comparable levels of nose poke investigation (Mann–Whitney: WT: *U* = 59.0, *p* = 0.65, *Pdzd8*^*tm1b*^: *U* = 28.0, *p* = 0.72). Social odor detection by *Pdzd8*^*tm1b*^ mice is intact, as head-fixed subjects displayed enhanced orofacial movement indicative of sniffing [47] when presented with female or male urine (Fig. 6C). *Pdzd8*^*tm1b*^ mice of both sexes are thus impaired in social odor discrimination.

### Brain morphological alterations in *Pdzd8*^*tm1b*^ mice

**Fig. 7:**
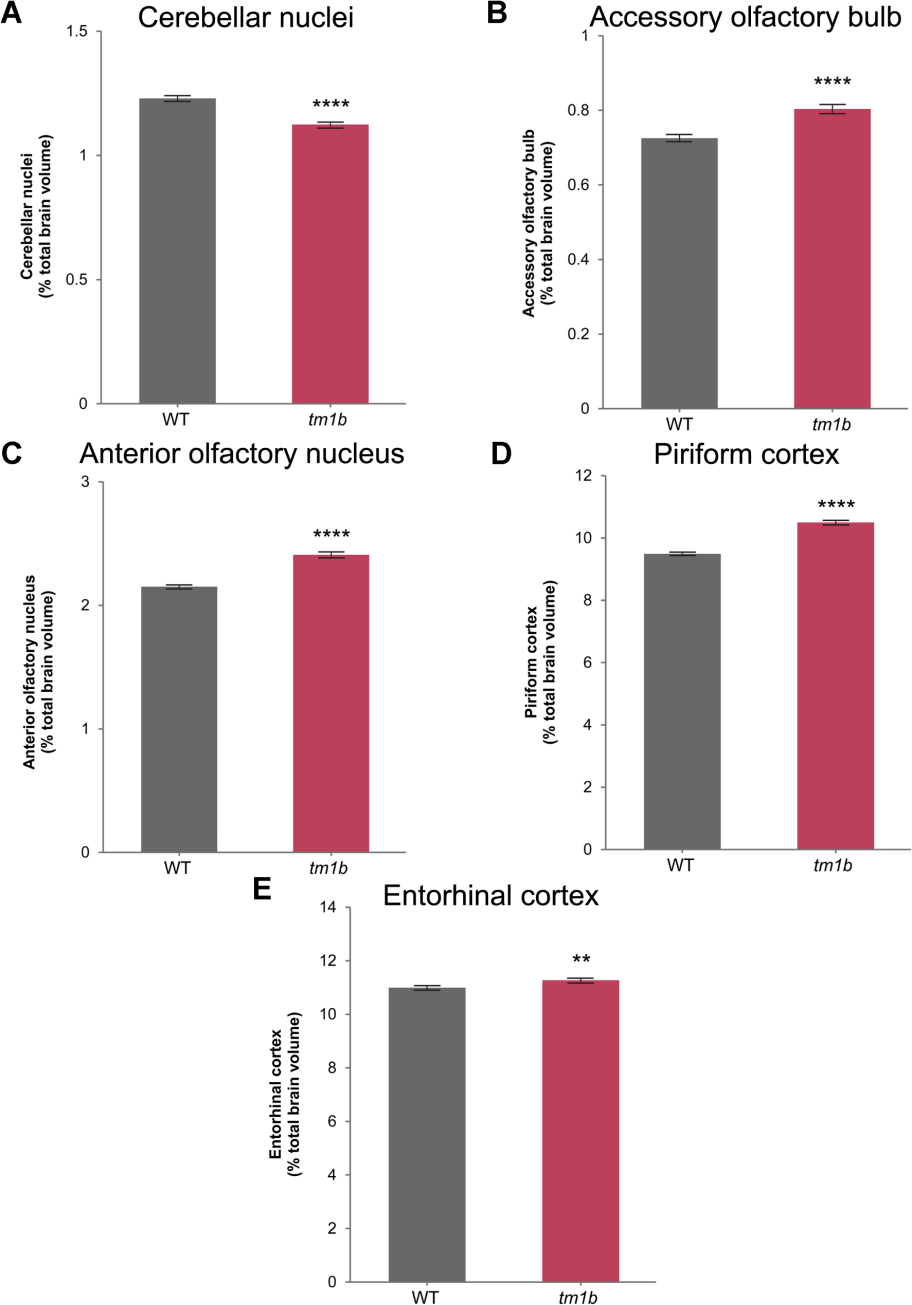
Relative (% total brain volume) volumetric differences in the cerebellar nuclei, accessory olfactory bulb, and components of the primary olfactory cortex in *Pdzd8*^*tm1b*^ mice (*n* = 32; 10♂, 22♀) and WT controls (*n* = 17; 7♂, 10♀) determined by high-resolution structural magnetic resonance imaging. **A** Cerebellar nuclei relative volume is decreased in *Pdzd8*^*tm1b*^ mice by 8.56 ± 0.79% (unpaired *t*-test: *t* = 7.83, *p* < 0.0001). **B** Accessory olfactory bulb relative volume is increased in *Pdzd8*^*tm1b*^ mice by 10.79 ± 1.38% (unpaired *t*-test: *t* = 6.18, *p* < 0.0001). **C** Anterior olfactory nucleus relative volume is increased in *Pdzd8*^*tm1b*^ mice by 11.99 ± 0.92% (unpaired *t*-test: *t* = 11.03, *p* < 0.0001). **D** Piriform cortex relative volume is increased in *Pdzd8*^*tm1b*^ mice by 10.58 ± 0.72% (unpaired *t*-test: *t* = 12.87, *p* < 0.0001). **E** Entorhinal cortex relative volume is increased in *Pdzd8*^*tm1b*^ mice by 2.57 ± 0.66% (unpaired *t*-test: *t* = 2.94, *p* = 0.005). *tm1b*, *Pdzd8*^*tm1b*^ homozygous; WT, wild-type. **p < 0.01, ****p < 0.0001 versus WT

We previously reported that MRI revealed brain structural alterations in *Pdzd8*^*tm1b*^ mice, including a decreased overall brain volume and increased relative volumes (in relation to the overall brain volume) of the OB, cerebellum, and hippocampus compared with WT littermate controls [2]. In light of the mild cerebellar hemispheric atrophy of the index case in family C (C.IV.1) and the social recognition and social odor discrimination deficits of *Pdzd8*^*tm1b*^ mice, we re-examined the murine MRI data for volumetric changes in several sub-regions that were not included in the original analysis. This revealed that the relative volume (% total brain volume) of the cerebellar nuclei (dendate nucleus, interposed nucleus, and fastigial nucleus), implicated in social behavior [48], is decreased in *Pdzd8*^*tm1b*^ mice (Fig. 7A). By contrast, the relative volumes of the accessory olfactory bulb (AOB), involved in processing social chemosensory information [49], and components of the primary olfactory cortex (anterior olfactory nucleus (AON), piriform cortex, and entorhinal cortex) [50, 51], are increased in *Pdzd8*^*tm1b*^ mice (Fig. 7B–D). The relative volumes of the dendate nucleus, interposed nucleus and fastigial nucleus individually, and significantly different absolute volumes (mm^3^) are shown in Additional file 4.

**Fig. 8:**
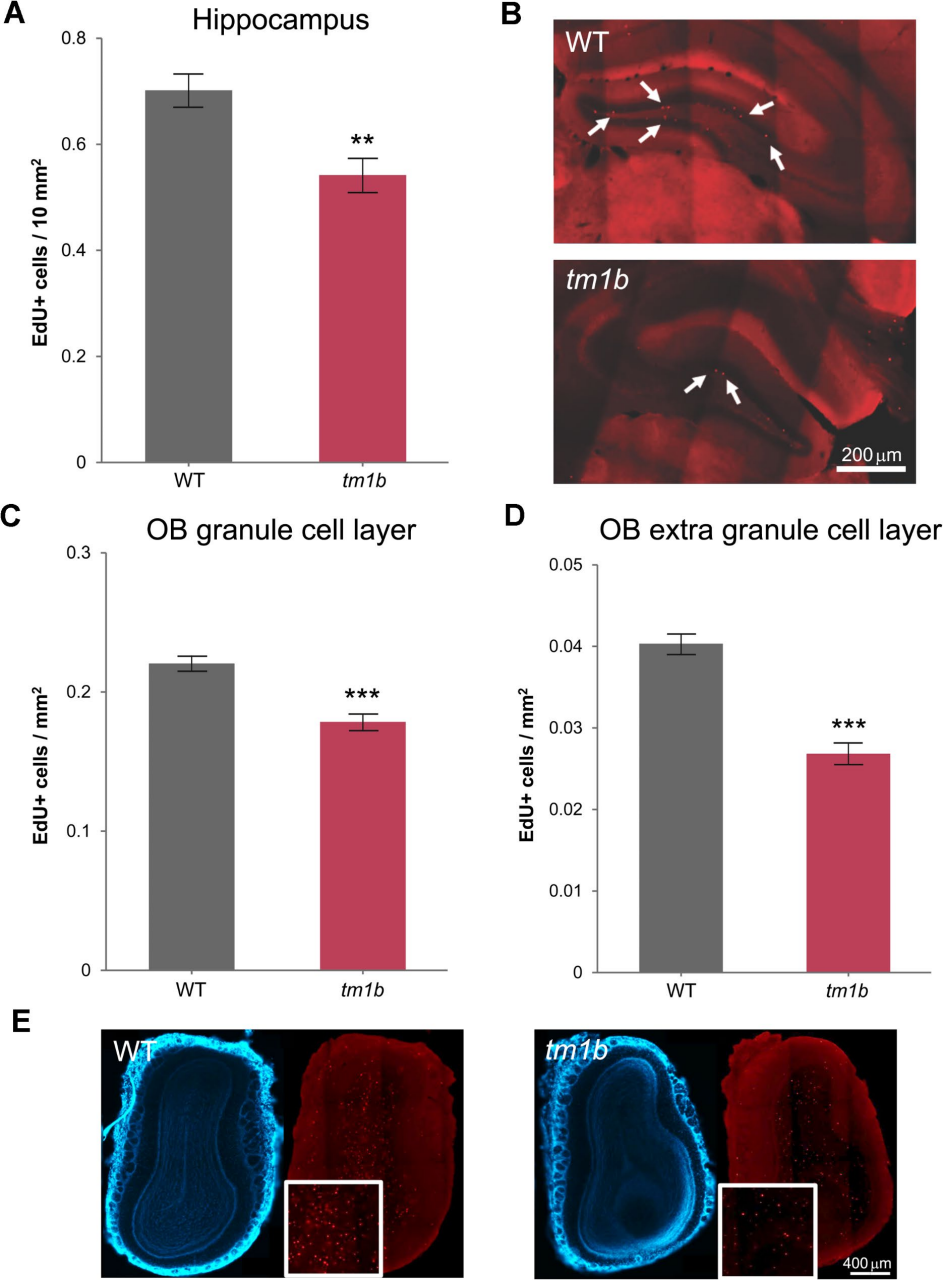
Decreased adult neurogenesis in *Pdzd8*^*tm1b*^ mice. **A** Number of EdU-positive cells relative to surface area (10 mm^2^) in sequential coronal sections of the hippocampus from *Pdzd8*^*tm1b*^ mice (*n* = 55 sections from *n* = 5♀ mice) and WT controls (*n* = 70 sections from *n* = 5♀ mice) (Mann–Whitney: *U* = 1,286, *p* < 0.0014). **B** Representative images of EdU staining in the hippocampus of WT control (*top*) and *Pdzd8*^*tm1b*^ mouse (*bottom*) with EdU-positive cells indicated by white arrows. The striations are artifacts caused by uneven illumination by the AxioScan Slide Scanner of the component image tiles that were assembled into the whole-section images. Scale bar: 200 μm. **C** Number of EdU-positive cells relative to surface area (mm^2^) in sequential coronal sections of the granule cell layer of the OB from *Pdzd8*^*tm1b*^ mice (*n* = 54 sections from *n* = 5♀ mice) and WT controls (*n* = 73 sections from *n* = 5♀ mice) (Mann–Whitney: *U* = 1,307, *p* = 0.0011). **D** Number of EdU-positive cells relative to surface area (mm^2^) in sequential coronal sections of the extra granule cell layer of the OB from *Pdzd8*^*tm1b*^ mice (*n* = 54 sections from *n* = 5♀ mice) and WT controls (*n* = 73 sections from *n* = 5♀ mice) (Mann–Whitney: *U* = 897, *p* < 0.0001). **E** Representative images of EdU staining in the OB of WT control (*left*) and *Pdzd8*^*tm1b*^ mouse (*right*) with white box showing EdU-positive cells in a zoomed-in area. Scale bar: 400 μm. +, positive; OB, olfactory bulb; *tm1b*, *Pdzd8*^*tm1b*^ homozygous; WT, wild-type

Considering the deficits in adult neurogenesis displayed by various murine models for autism [52-54], we examined *Pdzd8*^*tm1b*^ mice for alterations in adult neurogenesis in the hippocampus and OB, the main neurogenic regions of the adult brain [55]. This revealed that the density of EdU-labeled cells in both the hippocampus, predominantly in the dentate gyrus (Fig. 8A, B), and the OB, predominantly in the granule cell layer (Fig. 8C–E), is lower in *Pdzd8*^*tm1b*^ mice compared with WT littermate controls. *Pdzd8*^*tm1b*^ mice thus exhibit a reduction in adult neurogenesis, consistent with published observations in autism models [52-54].

**Fig. 9:**
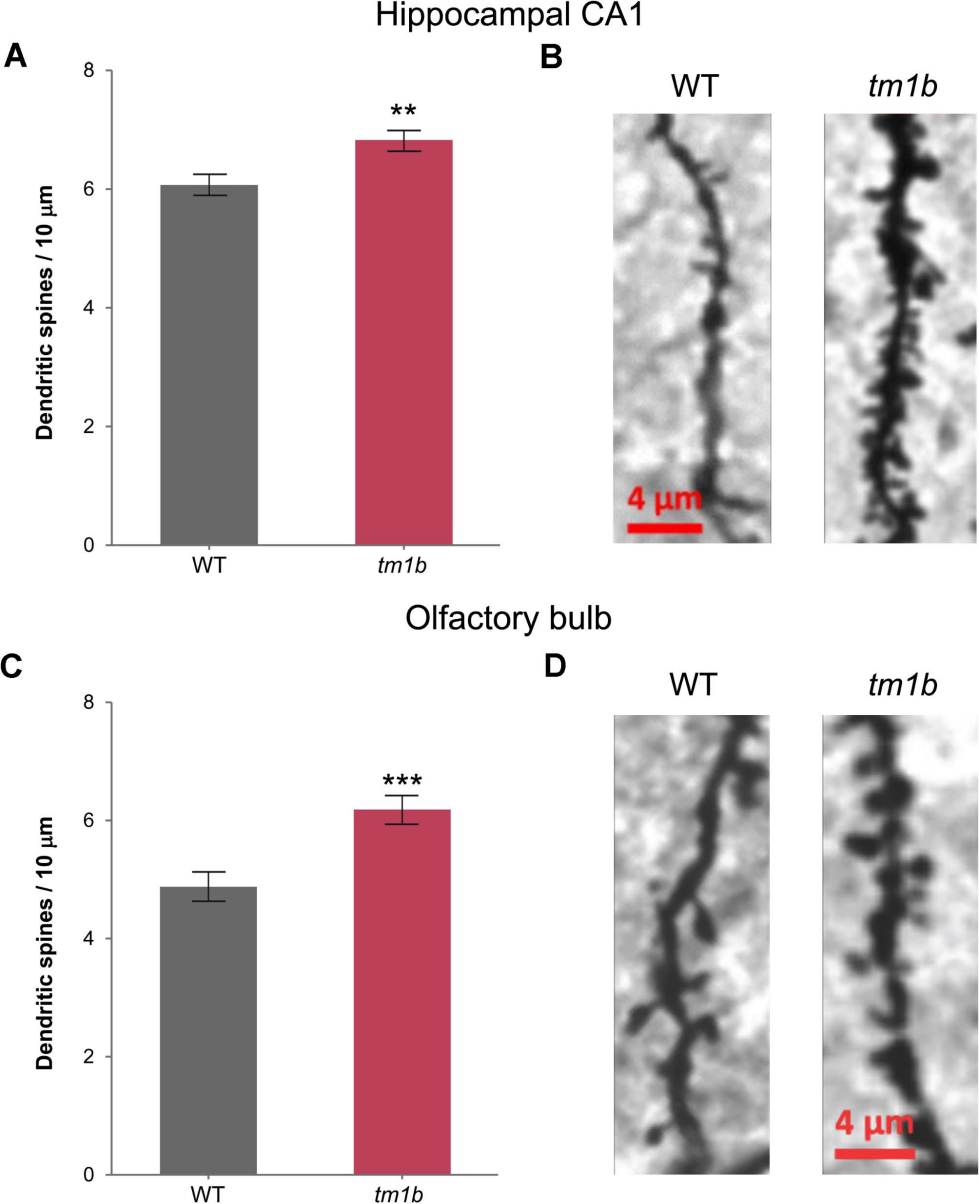
Greater density of dendritic spines in the hippocampal CA1 and the granule cell layer of the OB in *Pdzd8*^*tm1b*^ mice. **A** Density of dendritic spines in the hippocampal CA1 of *Pdzd8*^*tm1b*^ mice (*n* = 69 dendrites from *n* = 4♂ mice) and WT controls (*n* = 87 dendrites from *n* = 4♂ mice) (two-sample *t*-test: *t*(154) = 3.221, *p* = 0.0016). **B** Representative images of dendritic segment of Golgi–Cox-stained neurons in hippocampal CA1 of *Pdzd8*^*tm1b*^ mouse (*right*) and WT control (*left*). Scale bar: 4 μm. **C** The density of dendritic spines in the granule cell layer of the OB was higher (two-sample *t*-test with Welch’s correction: *t*(79.4) = 4.145, *p* = 0.000084) and less variable (Levene’s test, *p* = 0.017) in *Pdzd8*^*tm1b*^ mice (*n* = 27 dendrites from *n* = 4♂ mice) compared with WT controls (*n* = 57 dendrites from *n* = 4♂ mice). **D** Representative images of dendritic segment of Golgi–Cox-stained neurons in the granule cell layer of the OB of WT control (*left*) and *Pdzd8*^*tm1b*^ mouse (*right*). Scale bar: 4 μm. *tm1b*, *Pdzd8*^*tm1b*^ homozygous; WT, wild-type. **p < 0.01, ***p < 0.001 versus WT

As analysis of post-mortem brain samples has indicated higher dendritic spine densities in cortical neurons from ASD patients, most commonly those with lower levels of cognitive functioning [56, 57], we examined *Pdzd8*^*tm1b*^ mice for Golgi–Cox staining of dendritic spines. Compared with WT littermate controls, *Pdzd8*^*tm1b*^ mice displayed a greater density of dendritic spines in the hippocampal CA1 (Fig. 9A, B) and in the granule cell layer of the OB (Fig. 9C, D), but not in the suprapyramidal and infrapyramidal blades of the dentate gyrus or in the frontal association cortex (Additional file 5).

### ER stress and mitochondrial fusion markers are upregulated in *Pdzd8*^*tm1b*^ mice

ER stress leads to induction of the transcription factor ATF4 (activating transcription factor-4), triggering the expression of a raft of genes to restore ER function and maintain cell survival, including transcripts encoding the ER chaperone HSPA5 (heat shock protein family A member 5) [58]. In common with PDZD8, the mitochondrial membrane protein MFN2 (mitofusin-2) is a component of mitochondria–ER contact sites (MERCS) that provide a tethering force to ensure proximity and communication between the two organelles [59]. MFN2 is also one of three GTPases, along with MFN1 and OPA1 (optic atrophy-1), that serve to fuse mitochondria, whereas fission proteins such as FIS1 (fission-1) act to fragment mitochondria [60].

**Fig. 10:**
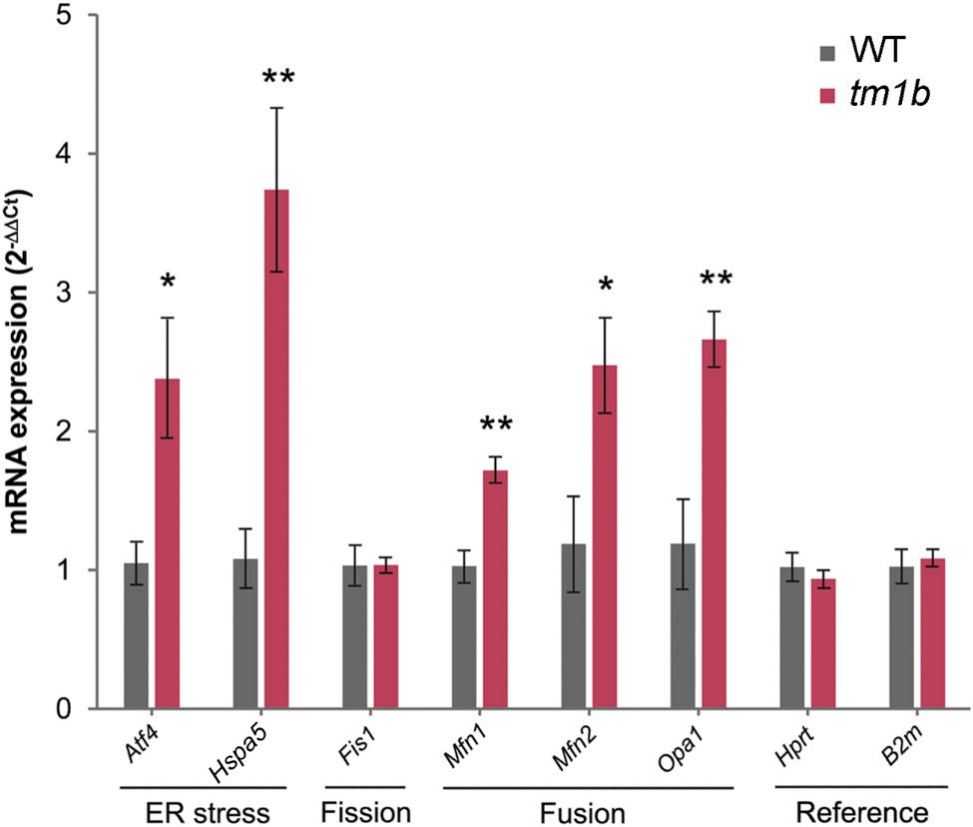
Altered mRNA expression in *Pdzd8*^*tm1b*^ mouse brain. Transcript levels of *Atf4* and *Hspa5* genes encoding ER stress markers, *Fis1* gene encoding a mitochondrial fission protein, *Mfn1*, *Mfn2* and *Opa1* genes encoding mitochondrial fusion markers, and the *Hprt* and *B2m* reference genes. Gene mRNA expression is presented as fold change ± SEM, calculated via the 2^-ΔΔCt^ method [30], relative to *Hprt* mRNA. Student’s *t*-test detected significant differences in male *Pdzd8*^*tm1b*^ homozygous mice versus WT controls (*n* = 5♂/genotype). Normalizing to the *B2m* reference gene gave similar results. *tm1b*, *Pdzd8*^*tm1b*^ homozygous; WT, wild-type. *p < 0.05, **p < 0.01 versus WT

To inspect whole brain samples from *Pdzd8*^*tm1b*^ mice and WT littermate controls (*n* = 5♂/genotype) for signs of ER stress, we employed qRT-PCR analysis. This revealed that mRNA transcript levels of the genes encoding the ER stress markers, ATF4 and HSPA5, and the mitochondrial fusion markers, MFN1, MFN2 and OPA1, are upregulated in *Pdzd8*^*tm1b*^ mice, but transcript levels of the FIS1 fission protein are unaltered (Fig. 10). *Pdzd8*^*tm1b*^ mice thus show evidence of increased ER stress and mitochondrial fusion in the brain.

## Discussion

We have identified a homozygous PTC variant (p.Q30*) in *PDZD8* that cosegregates with syndromic ID in a third family (family C). Family C is of Afghan origin, whereas family A is from Oman and family B is from the United Arab Emirates, all countries with high rates (> 20%) of consanguineous marriage [61]. All three IDDADF families have *PDZD8* PTC homozygosity and first cousin marriage in common. Autosomal recessive variants, such as *PDZD8* PTCs, are known to play a significant role in ID in populations with frequent parental consanguinity [62]. The two affected siblings in family C represent a 50% increase in known IDDADF cases. Unlike *PDZD8* variant p.(S733*) in family A and p.(Y298*) in family B, p.(Q30*) in family C is present in gnomAD version 4.1.0, in a single heterozygous non-Finnish European female not ascertained from the UK Biobank [31].

The identification of six IDDADF cases (4 male and 2 female), with three different *PDZD8* PTC variants and ethnic origins, permits the identification of a core clinical phenotype affecting all cases, consisting of developmental delay, ID, autism, and facial dysmorphism (Table 1). This knowledge will facilitate the genetic diagnosis of other patients. Five of the six IDDADF cases, including both affected siblings in family C, additionally have myasthenia. ID and cognitive impairment were the focus of our previous study [2], but herein we turned our attention to autistic behavior and metabolic phenotypes.

Among the previously published IDDADF cases [2], the one female and two male affected siblings in family A were all diagnosed with ASD. Examination of the female included ADOS-2 assessment [16], which confirmed the ASD diagnosis (Abeer Al Sayegh, personal communications). The affected male in family B exhibits autistic behavior in the form of poor eye contact, echolalia, hand flapping, and jumping whenever excited. However, after two years of intensive rehabilitation, he did not meet the full diagnostic criteria for ASD when evaluated using CARS2 and the Gilliam Autism Rating Scale, 3rd edition (GARS-3) [63] (Aisha Al Shamsi, personal communications).

The manifestation of autism in all six known IDDADF cases, with ASD diagnosed in five cases (83%) including both females, suggests that this trait may be fully penetrant in *PDZD8* PTC variant homozygotes regardless of sex. By comparison, ASD is diagnosed in 9–30% of all ID cases [64-67] and at an equal male-to-female ratio in non–X-linked genetic syndromes (syndromic autism), as opposed to idiopathic autism, which occurs 4–5 times more frequently in males than in females [68].

Despite ADHD affecting only two of the six IDDADF cases to date [2] – comparable with ADHD comorbidity rates of 15–20% across ID cases [69, 70] – locomotor hyperactivity during the dark phase was the most striking behavior exhibited by *Pdzd8*^*tm1b*^ mice singly-housed in metabolic cages. In another PDZD8-deficient mouse line (*Pdzd8*^*em1Kei*^), *Pdzd8* exon 1, containing the start codon, is deleted on a C57BL/6J genetic background [5]. Homozygous *Pdzd8*^*em1Kei*^ mice showed increased locomotor activity over 7 days in the home cage when males of the same genotype were housed in pairs, although the data do not distinguish between the lights-on and dark phases [71].

*Pdzd8*^*em1Kei*^ mice of an undefined sex exhibited a ∼2–3-fold increase in levels of cholesteryl esters, but not other lipids, in the basal ganglia compared with WT controls at 3–7 months of age [33]. This effect was less pronounced in the cortex (∼0.5-fold increase) despite a similar abundance of *Pdzd8* mRNA in both brain regions [33]. The effect of an accumulation of cholesteryl esters on the function of the basal ganglia is unknown. Mice with basal ganglia dysfunction induced by bilateral elimination of cortico-subthalamic inputs exhibited locomotor hyperactivity in an open field test [72], whereas male *Pdzd8*^*em1Kei*^ mice showed unaltered levels of locomotor activity during open field testing [71].

We previously observed that *Pdzd8*^*tm1b*^ mice show elevated levels of stereotypical motor behavior (repetitive hindlimb jumping) in home cages without a running wheel [2]. As a repetitive, monotonous pattern of movement, the excessive wheel running of *Pdzd8*^*tm1b*^ mice might be re-directed stereotypic behavior, like that reported for African striped mice [73] and TgCRND8 transgenic mice [74] that show locomotor stereotypy. In the TgCRND8 model for Alzheimer’s disease, access to a running wheel led to a strong reduction in the amount of stereotypic behavior (including jumping) and a concomitant inverse correlation between wheel-running and stereotypic behavior, but it had no effect on cognitive or neuropathological parameters [74]. Similarly, the presence of a free-spinning running wheel, but not a fixed (non-rotating) one, reduced stereotypical behavior in the C57BL/6J and CD-1 strains [75, 76]. Voluntary wheel running was also shown to reverse a range of behavioral abnormalities (sociability, self-grooming, and anxiety) in mice with neurodevelopmental impairments induced by maternal immune activation [77]. It remains to be determined whether voluntary wheel running has similar ameliorating effects on the behavioral abnormalities of *Pdzd8*^*tm1b*^ mice.

Despite the lower body weight of *Pdzd8*^*tm1b*^ mice [2], they consume the same amount of food as WT controls, likely due to their elevated locomotor activity and metabolic rate. The locomotor activity was increased only in the dark phase, when nocturnal mice are more active [38], but the metabolic rate was increased across the light/dark cycle, including the lights-on phase when *Pdzd8*^*tm1b*^ mice were resting. This may explain why male *Pdzd8*^*tm1b*^ mice have decreased plasma triglyceride levels.

Since functional PDZD8 is required for glutaminolysis in response to hypoglycemia [10], it is plausible that the PDZD8 deficiency and locomotor hyperactivity of *Pdzd8*^*tm1b*^ mice require them to obtain proportionately more fuel from the diet in order to meet their energy demands. A recent study of C57BL/6J mice fed a high-fat diet (HFD) showed that type II diabetes (T2D)-related phenotypes, including insulin resistance and pancreatic β-cell death, are accompanied by upregulation of *Pdzd8* mRNA levels in pancreatic islet tissue, but these effects were alleviated by knockdown of *Pdzd8* via systemic AAV9-mediated shRNA [78]. Given that the extension of healthspan and lifespan induced by calorie restriction requires functional PDZD8 [11], the effects on metabolic health of a reduction in PDZD8 levels may be detrimental under caloric restriction but beneficial under caloric excess.

In the reciprocal social interaction test and in the first trial of the three-chamber social approach test, female *Pdzd8*^*tm1b*^ mice demonstrated unaltered sociability toward an unfamiliar female C57BL/6J mouse. However, in the second trial of the social approach test, they demonstrated a reduced preference for social novelty compared with WT controls, suggesting deficient social recognition of familiar versus novel mice. In comparison, male *Pdzd8*^*em1Kei*^ mice showed an unaltered duration of interaction with an unfamiliar male of the same genotype (*Pdzd8*^*em1Kei*^–*Pdzd8*^*em1Kei*^ versus WT–WT), and unaltered levels of both sociability and preference for social novelty in a three-chamber social approach test [71]. The reasons for the apparent differences in social behavior between the two *Pdzd8* mouse lines are unknown but may be related to disparity in the genetic background (C57BL/6NTac versus C57BL/6J substrain) [79-82] or the sex [83] of the mice tested. A comparative summary of ASD/ADHD-relevant phenotypes of the *Pdzd8*^*tm1b*^ and *Pdzd8*^*em1Kei*^ mouse lines is given in Table 2.

**Table 2:** Comparative summary of ASD/ADHD-relevant phenotypes of the *Pdzd8*^*tm1b*^ and *Pdzd8*^*em1Kei*^ mouse lines

Characteristic	Pdzd8^tm1b^		Pdzd8^em1Kei^		References
*Pdzd8* gene disruption	Replacement of exon 3 by *lacZ* cassette		Deletion of exon 1		[2, 5]
Effect on PDZD8 protein	p.(F333Nfs1*)		Protein null		
Genetic background	C57BL/6NTac		C57BL/6J		[2, 5]
Zygosity	Homozygous		Homozygous		
Sex	♂	♀	♂	♀	
Repetitive jumping	>>	>>	–	–	[2]
Locomotor activity					
Open field	>	>	ns	–	[2, 27]
In-cage ambulation	> (dark)	–	>	–	Figure 3A, B, [71]
Wheel running	>> (dark)	–	–	–	Figure 3C, D
Elevated plus maze					[2, 71]
Open arm entries	>	>	>>	–	
Open arm time	ns	ns	>>	–	
Social interaction time with C57BL/6J juvenile ♀	–	ns	–	–	Figure 5A
with same genotype adult ♂	–	–	ns	–	[71]
3-chamber social approach					Figure 5D, [71]
Sociability	–	ns	ns	–	
Social novelty preference	–	<	ns	–	
Social odor discrimination	<<	<<	–	–	Figure 6

–, not reported; <, lower than WT; <<, much lower than WT; >, greater than WT; >>, much greater than WT; (dark), dark phase only; ns, not significantly different

In the cross-habituation assay [24], *Pdzd8*^*tm1b*^ mice of both sexes were unable to discriminate between female and male urine, suggesting an impairment in social odor discrimination like that exhibited by the BTBR model [46]. As head-fixed *Pdzd8*^*tm1b*^ mice showed unaltered odor detection when presented with female or male urine, the social odor discrimination deficit cannot be attributed to a diminished ability to detect social odors. We previously observed a similar deficit in social discrimination with intact olfaction in a mouse model for autism-associated 2p16.3 deletion [23].

Brain MRI revealed mild cerebellar hemispheric atrophy in the index case in family C (C.IV.1). Although we previously reported an increased relative volume of the whole cerebellum in *Pdzd8*^*tm1b*^ mice [2], the present study found that the relative volume of the cerebellar nuclei is decreased. Chemogenetic inhibition of subthalamic zona incerta neurons that receive projections from cerebellar nuclei has been shown to rescue a social novelty preference deficiency in the *Nlgn3*^R451C^ mouse model for X-linked autism [48].

The relative volumes of the AOB, AON, piriform cortex and entorhinal cortex are increased in *Pdzd8*^*tm1b*^ mice. Olfactory deprivation has been shown to reduce the volume of the AON, particularly later-developing subdivisions that receive the bulk of projections from the OB [84]. Experimental activation of the AON was shown to reduce olfactory sensitivity and impair recognition of a novel conspecific [85], and to suppress odor responses regardless of odor identity or concentration [86], suggesting an inhibitory effect of the AON on olfaction-dependent behaviors. Consequently, it is tempting to hypothesize that an enlarged AON exerting greater inhibition may contribute to the social recognition and social odor discrimination deficits of *Pdzd8*^*tm1b*^ mice. The AOB is absent in humans and other higher primates [50].

The decreased neurogenesis in the hippocampus and OB of adult *Pdzd8*^*tm1b*^ mice, as assessed by EdU labeling, is comparable with deficits in adult neurogenesis exhibited by the BTBR, VPA-induced, and *Nlgn3*^R451C^ mouse models for autism [52-54]. However, whether the deficient neurogenesis contributes to the social odor discrimination deficit of *Pdzd8*^*tm1b*^ mice remains to be determined.

The increased density of dendritic spines in the hippocampal CA1 and OB of *Pdzd8*^*tm1b*^ mice, as assessed by Golgi–Cox staining, is similar to that observed in ASD subjects with severe ID, but not in ASD subjects with either mild or no ID [56, 57]. Within the OB, granule cells are the only cell type to possess spines and are important for discriminating between odors [87]. Granule cells receive extensive centrifugal inputs, including inputs from the AON that are thought to be responsible for social odor discrimination [88]. The AON is enlarged and the spine density of granule cells is elevated in *Pdzd8*^*tm1b*^ mice, yet the variation in spine density is lower. This may reflect a defect in homeostatic synaptic scaling, whereby the set point for synaptic density is set close to saturation, thus limiting the scope for learning to discriminate relative social cues. Similarly increased numbers of dendritic spines have been found in brain samples from patients and mouse models with fragile X syndrome (FXS) [89-91], the most frequent monogenic cause of ID, which is often accompanied by autistic behavior [92]. These independent lines of evidence suggest that dendritic spine abnormalities may impair the processing of socially relevant information. However, owing to a lack of postmortem samples, the density of dendritic spines in brains of *PDZD8*-related IDDADF patients is currently unknown.

Our mRNA expression analysis provides evidence of transcriptional upregulation of ER stress (ATF4 and HSPA5) and mitochondrial fusion (MFN1, MFN2 and OPA1) markers in brain tissue of *Pdzd8*^*tm1b*^ mice. Upregulation of *ATF4* and some of its target genes, including *HSPA5*, was previously observed in wild-type HeLa cells exposed to the ER stressor tunicamycin [93]. Several mitochondrial stressors induced the same ATF4-dependent transcriptional stress response but had no effect on *HSPA5* transcript levels or in *ATF4* knockout HeLa cells [93]. While the mitochondrial and ER stress responses both rely on ATF4 signaling [93], the upregulation of both *Atf4* and *Hspa5* in *Pdzd8*^*tm1b*^ mouse brain is more characteristic of the latter.

Increased mRNA expression of ER stress-related genes, including *ATF4*, has previously been observed in the middle frontal gyrus of ASD subjects, and this change was positively associated with the severity of stereotyped autistic behavior [94]. Recently, a systematic analysis of mRNA expression profiles from the Gene Expression Omnibus database revealed the differential expression of ER stress regulators in ASD subjects versus controls [95]. Signs of ER stress in the brain have also been observed in 1-day-old pups of a mouse model for autism induced by in utero exposure to VPA [96], an established environmental risk factor for ASD [97].

In the livers of both genetic (*ob*/*ob*; leptin deficient) and HFD-induced mouse models for obesity, mitochondria–ER contact was greater than in lean controls, resulting in increased Ca^2+^ flux from the ER to mitochondria, mitochondrial Ca^2+^ overload, compromised mitochondrial oxidative capacity, and augmented oxidative stress [98]. In pancreatic islet tissue of a HFD-induced mouse model for T2D, increased levels of ER stress markers and signs of mitochondrial dysfunction (e.g., oxidative stress, ATP depletion), together with enhanced *Pdzd8* transcript and mitochondria–ER contact levels, were alleviated by *Pdzd8* knockdown [78]. These findings suggest that the obesogenic effects of leptin deficiency and HFD feeding may be attenuated by a reduction in PDZD8 levels, although the hepatic lipid content of 3-month-old *Pdzd8*^*em1Kei*^ mice fed either a normal diet or a high-fat diet was not substantially different from that of WT controls [33].

The upregulation of mitochondrial fusion markers in *Pdzd8*^*tm1b*^ mice that show signs of ER stress suggests that PDZD8 deficiency affects mitochondrial dynamics. In support, knockdown of *PDZD8* using siRNA has been shown to increase mitochondrial volume and oxidative stress, and to suppress mitochondrial respiration via mitochondrial Fe^2+^ accumulation, in human gastric cancer cell lines [99]. Mitochondrial fusion events are important under stress conditions, where promoting ATP production is crucial for cell survival [100, 101]. As mitochondrial Ca^2+^ uptake, supported by PDZD8, stimulates ATP production [102], and mitochondrial fusion leads to an increase in ATP production [103], increased mitochondrial fusion in *Pdzd8*^*tm1b*^ mice may reflect an adaptive response to counteract a potential deficit in mitochondrial Ca^2+^ uptake caused by PDZD8 deficiency, although testing this hypothesis would require additional studies.

### Limitations

It is important to acknowledge that this study has several limitations. Firstly, the observations on the incidence of clinical features in IDDADF are based on only six known cases of *PDZD8* PTC homozygosity. However, cross-species support is provided by PDZD8-deficient *Pdzd8*^*tm1b*^ mice that exhibit phenotypes comparable to ID and autism [2].

Secondly, some mouse experiments in the study did not use balanced numbers of males and females, for various reasons. To reduce non-genotypic variability, brain samples from single sex groups were used for the ex vivo assessment of neurogenesis (♀ only), dendritic spine density (♂ only), and ER stress and mitochondrial fusion markers (♂ only). Due to limited availability of metabolic cages, only male mice were assessed in them. Juvenile social interaction testing was restricted to females to avoid aggressive interactions associated with males [104]. Owing to a technical problem with most of the video recordings of *Pdzd8*^*tm1b*^ males, three-chamber social approach testing was limited to females. Nonetheless, *Pdzd8*^*tm1b*^ mice of both sexes showed impaired social odor discrimination in the cross-habituation assay. Previous behavioral assessment of PDZD8 deficiency in the *Pdzd8*^*em1Kei*^ mouse line used males only [71]. Testing of both sexes should be prioritised in future studies.

Thirdly, the quantification of ER stress and mitochondrial fusion markers did not extend beyond qRT-PCR analysis of mRNA levels, an approach previously used to quantify ER stress markers in human cell lines [93] and brain tissue [94, 95].

## Conclusions

In summation, this study identifies a third family with IDDADF caused by biallelic disruption of *PDZD8*, thereby permitting the identification of a core clinical phenotype including autism. The *Pdzd8*^*tm1b*^ mouse line exhibits impairments in social recognition and social odor discrimination, along with alterations in locomotor activity, brain structure (cerebellar nuclei, AOB, and primary olfactory cortex volumes), dendritic spine density, and adult neurogenesis. Autistic behavior is thus a common outcome of disruption of PDZD8 in humans and mice. The physical and metabolic abnormalities also exhibited by *Pdzd8*^*tm1b*^ mice suggest that the range of comorbidities associated with PDZD8 deficiency may be wider than presently recognized.

## Electronic supplementary material

Below is the link to the electronic supplementary material.

Supplementary Material 1

Supplementary Material 2

Supplementary Material 3

Supplementary Material 4

Supplementary Material 5

## Data Availability

The data that support the findings of this study are available from the corresponding author upon reasonable request.

## Abbreviations

♀: Female mice
♂: Male mice
aa: Amino acid
AAV9: Adeno-associated virus 9
ADHD: Attention-deficit/hyperactivity disorder
ADOS-2: Autism Diagnostic Observation Schedule, 2nd edition
AMPK: AMP-activated protein kinase
ANOVA: Analysis of variance
AOB: Accessory olfactory bulb
AON: Anterior olfactory nucleus
ASD: Autism spectrum disorder
ATF4: Activating transcription factor-4
ATP: Adenosine triphosphate
B6N: C57BL/6NTac
CA1: Cornu ammonis-1
CADD: Combined Annotation-Dependent Depletion
CARS2: Childhood Autism Rating Scale, 2nd edition
cDNA: Complementary deoxyribonucleic acid
CLAMS: Comprehensive Lab Animal Monitoring System
CNV: Copy number variant
C-terminal: Carboxy-terminal
dB: Decibels
DNA: Deoxyribonucleic acid
DSM-5: Diagnostic and Statistical Manual of Mental Disorders, 5th edition
EdU: 5-ethynyl-2′-deoxyuridine
ER: Endoplasmic reticulum
FIS1: Fission-1
GARS-3: Gilliam Autism Rating Scale, 3rd edition
Hz: Hertz
HFD: High-fat diet
HSPA5: Heat shock protein family A member 5
ID: Intellectual disability
IDDADF: Intellectual developmental disorder with autism and dysmorphic facies
IMPC: International Mouse Phenotyping Consortium
IMPReSS: International Mouse Phenotyping Resource of Standardised Screens
IQ: Intelligence quotient
IsoA: Isoamyl acetate
Mat: Maternal
MERCS: Mitochondria–ER contact sites
MFN1: Mitofusin-1
MFN2: Mitofusin-2
MRC: Medical Research Council
MRI: Magnetic resonance imaging
mRNA: Messenger ribonucleic acid
mtDNA: Mitochondrial DNA
OB: Olfactory bulb
OMIM: Online Mendelian Inheritance in Man
OPA1: Optic atrophy-1
Pat: Paternal
PBS: Phosphate-buffered saline
PCR: Polymerase chain reaction
PDZD8: PDZ domain-containing protein 8
*Pdzd8*^*tm1b*^: *Pdzd8* ^*tm1b(EUCOMM)Wtsi*^
*Pdzd8*^*tm1b(EUCOMM)Wtsi*^: PDZ domain containing 8; targeted mutation 1b, Wellcome Trust Sanger Institute
PTC: Premature termination codon
PROBES: Poking registered olfactory behaviour evaluation system
qRT-PCR: Quantitative reverse transcriptase polymerase chain reaction
Urine(f): Urine from female mice
Urine(m): Urine from male mice
RER: Respiratory exchange ratio
RNA: Ribonucleic acid
SCCM: Standard cubic centimetres per minute
shRNA: Short hairpin RNA
siRNA: Short interfering RNA
T2D: Type II diabetes
*tm1b*: *Pdzd8*^*tm1b*^ homozygous
VPA: Valproic acid
WES: Whole-exome sequencing
WT: Wild-type
