# Supplementary material for: Autistic behavior is a common outcome of biallelic disruption of PDZD8 in humans and mice
Source: Mol Autism. 2025 Feb 27;16:14. doi: 10.1186/s13229-025-00650-8 (PMC11866840; doi:10.1186/s13229-025-00650-8)
Supplement: Supplementary file 1 — Supplementary Material 1 [file 13229_2025_650_MOESM1_ESM.pdf]

Additional File 1

Childhood Autism Rating Scale (CARS2) scores for patient C.IV.1 – 13 years-old (male)

**Domains**	**Scores**
Relationship to people	3.5
Imitation	2
Emotional response	3
Body use	3.5
Object use	3.5
Adaptation to change	2.5
Visual response	3.5
Listening response	1.5
Taste, smell, and touch response and use	4
Fear or nervousness	3
Verbal Communication	3
Nonverbal Communication	3
Activity Level	3.5
Level and consistency of intellectual response	2
General Impressions	3.5
**Total raw score**	45 (= severe symptoms of autism spectrum disorder)

1 indicates that the child's behavior is within normal limits for a person of the same age; **1.5** indicates that the child's behavior is very slightly abnormal for the age; **2** indicates that the child's behavior is slightly abnormal compared to a subject of the same age; **2.5** indicates that the child's behavior is slightly to moderately abnormal for their age; **3** indicates that the child's behavior is moderately abnormal for this age; **3.5** indicates that the child's behavior is moderately to severely abnormal for the age; **4** indicates that the child's behavior is severely abnormal for that age. The diagnostic category is determined based on the child's total score and the number of scales in which he/she obtained a score of 3 or higher. A total raw score > 35 is the cut-off value for severe symptoms for patients >13 years old.

Vineland Adaptive Behavior Scales, 2nd edition (Vineland-II) scores for patient C.IV.1 – 13 years old (male)

Sub-domain	Raw score obtained/expected at his age	Percentile	Age conversion
**Communication**		<P1	
Receptive	9/39		<1 year
Expressive	7/106		
Written	0/44		
**Daily living skills**		<P1	
Personal	2/73		<1 year
Domestic	0/32		
Community	0.65		
**Socialization**		<P1	
Interpersonal relationships	11/68		<1 year
Play and leisure time	0/56		
Coping skills	0/53		
**Motor skills**		<P1	
Gross motor	Between 44 and 58/79		Between 1 year, 5 months and 2 years 7 months
Fine motor	Between 24 and 30/67		Between 1 year, 9 months and 2 years, 7 months

The Vineland-II questionnaire indicates a severe developmental delay, with significant impairments across all areas of adaptive skills in patient C.IV.1. In the absence of stimulation, patient C.IV.1 displays minimal spontaneous interest, suggesting a profound intellectual disability. Additionally, the presence of relational and communication difficulties, repetitive behaviors, and hypersensitivity to noise points toward a diagnosis of autism spectrum disorder.

Psychoeducational Profile, 3rd edition (PEP-3) scores for patient C.IV.2 – 8 years and 3 months old (female)

Sub-tests	Raw Score	Percentile	Difficulty level
**Communication**		**P1**	**Severe**
Cognitive verbal/preverbal	23	P15	Severe
Expressive language	0	P5	Severe
Receptive language	3	P10	Severe
**Motor**		**P7**	**Severe**
Fine motor	22	P10	Severe
Gross motor	22	P10	Severe
Visual-motor imitation	7	P10	Severe
**Maladaptive behaviors**		**P4**	**Severe**
Affective expression	5	P5	Severe
Social reciprocity	2	P4	Severe
Characteristic motor behaviors	16	P10	Severe
Characteristic verbal behaviors	4	P6	Severe

The PEP-3 assesses the skills and behaviors of children with autism and communicative disabilities. The evaluation revealed a severe developmental delay, particularly pronounced in the three domains of communication. In contrast, gross motor skills appear to be relatively less impacted. Maladaptive behaviors commonly associated with autism, such as difficulties in social reciprocity, impaired affective expression, language delays, echolalia, and stereotypies, were notably present.

Autism Diagnostic Observation Schedule, 2nd edition (ADOS-2), Module 1 (few to no words); scores for patient C.IV.2 – 8 years and 3 months old (female)

Items	Scores
**Social affect (SA)**	**17**
Frequency of spontaneous vocalization directed to others	2
Gestures	1
Unusual eye contact	2
Facial expressions directed to others	2
Integration of gaze and other behaviors during social overtures	2
Shared enjoyment in interaction	1
Showing	2
Spontaneous initiation of joint attention	2
Response to joint attention	2
Quality of social overtures	1
**Restricted and repetitive behavior (RRB)**	**8**
Intonation of vocalizations or verbalizations	2
Unusual sensory interest in play material/person	2
Hand and finger and other complex mannerisms	2
Unusually repetitive interests or stereotyped behaviors	2
**Overall score (SA + RRB)**	**25**
ADOS-2 classification	Autism spectrum disorder
Level of autism spectrum-related symptoms	High

Based on coding conventions for the item scores (i.e., a score of 0 indicates typical or expected skills, while a score of 2 indicates behavior that is either atypical or notably absent), the patient exhibits severe symptoms across all domains of autism. The global score exceeds the diagnostic cut-off value (>16), supporting a diagnosis of autism spectrum disorder.
