# Supplementary material for: Autistic behavior is a common outcome of biallelic disruption of PDZD8 in humans and mice
Source: Mol Autism. 2025 Feb 27;16:14. doi: 10.1186/s13229-025-00650-8 (PMC11866840; doi:10.1186/s13229-025-00650-8)
Supplement: Supplementary file 2 — Supplementary Material 2 [file 13229_2025_650_MOESM2_ESM.pdf]

Additional File 2

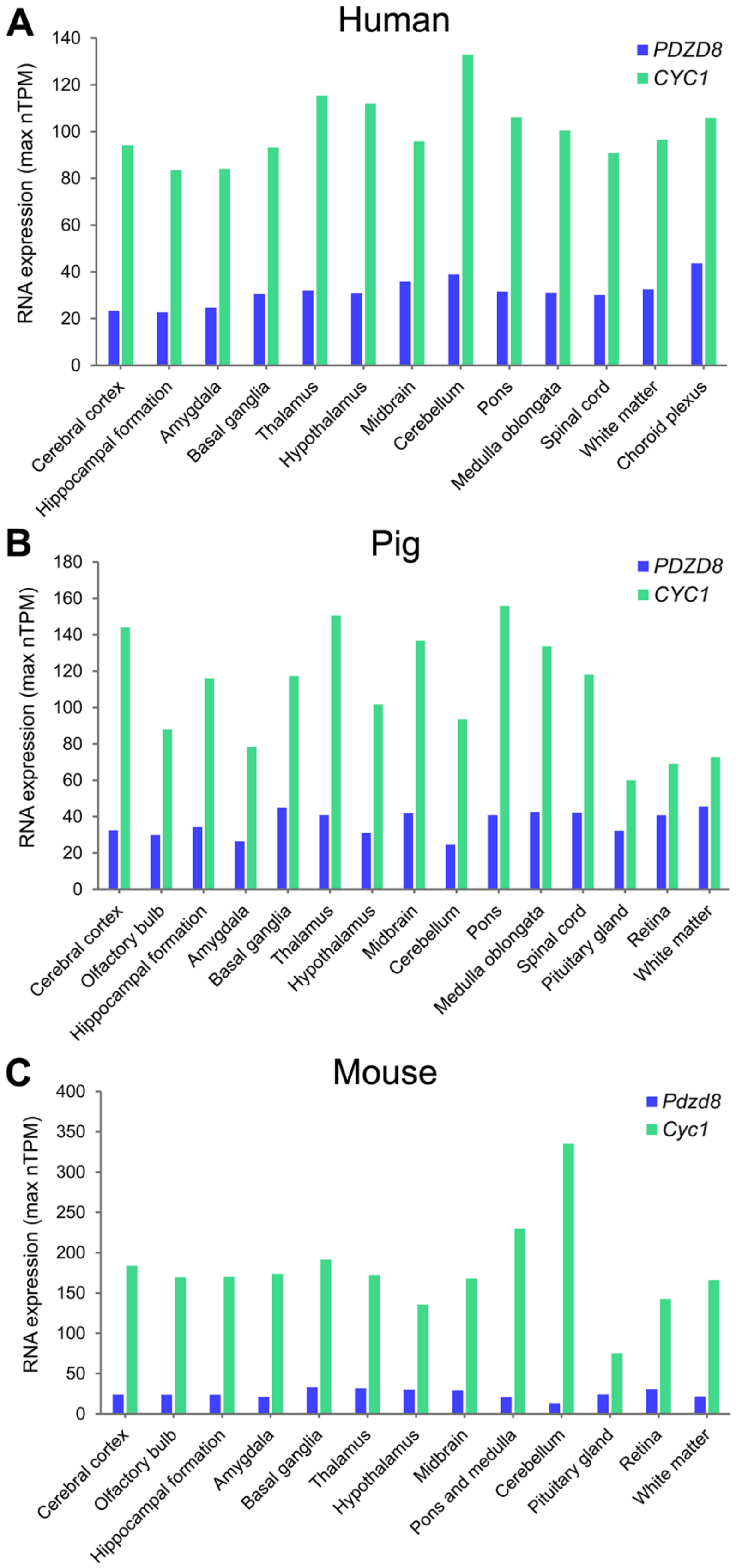

PDZD8 mRNA expression in regions of the mammalian brain. RNA-seq expression data from the Human Protein Atlas for PDZD8 and CYC1 (reference gene) in regions of the human (**A**), pig (**B**) and mouse (**C**) brain, presented as maximum normalized transcripts per million (max nTPM) [32].
