# Supplementary material for: Autistic behavior is a common outcome of biallelic disruption of PDZD8 in humans and mice
Source: Mol Autism. 2025 Feb 27;16:14. doi: 10.1186/s13229-025-00650-8 (PMC11866840; doi:10.1186/s13229-025-00650-8)
Supplement: Supplementary file 3 — Supplementary Material 3 [file 13229_2025_650_MOESM3_ESM.pdf]

Additional File 3

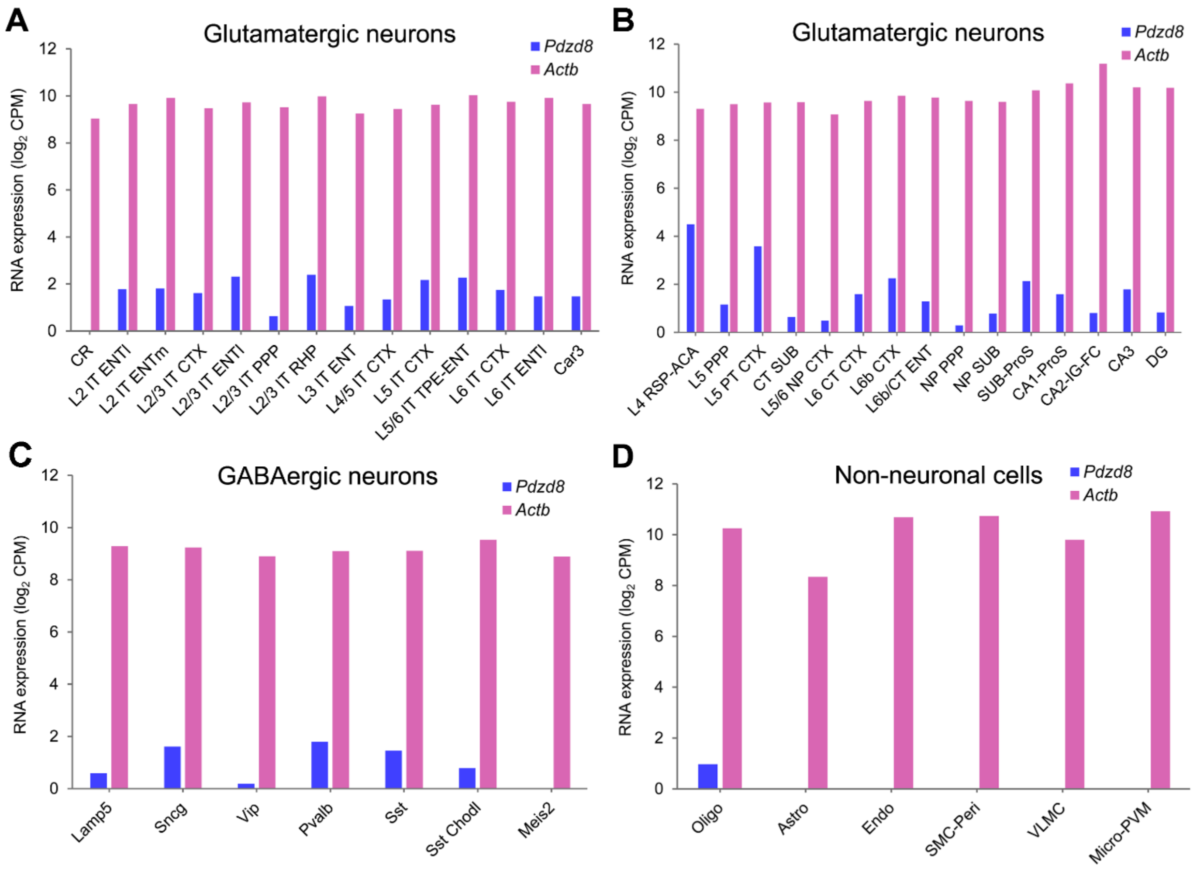

Pdzd8 mRNA expression in different cell types in adult mouse cerebral cortex and hippocampus. Single-cell RNA-seq data from the Allen Cell Types Database for *Pdzd8* and *Actb* (reference gene) among 42 types of glutamatergic (**A**, **B**) and GABAergic (**C**) neurons and non-neuronal cells (**D**) in cerebral cortex and hippocampal formation from adult (postnatal day P53-P59) mice, presented as trimmed mean log_2 CPM (counts per million reads mapped), averaged after excluding the 25% highest and 25% lowest expression values [34].
