# Supplementary material for: Autistic behavior is a common outcome of biallelic disruption of PDZD8 in humans and mice
Source: Mol Autism. 2025 Feb 27;16:14. doi: 10.1186/s13229-025-00650-8 (PMC11866840; doi:10.1186/s13229-025-00650-8)
Supplement: Supplementary file 4 — Supplementary Material 4 [file 13229_2025_650_MOESM4_ESM.pdf]

**Additional File 4**

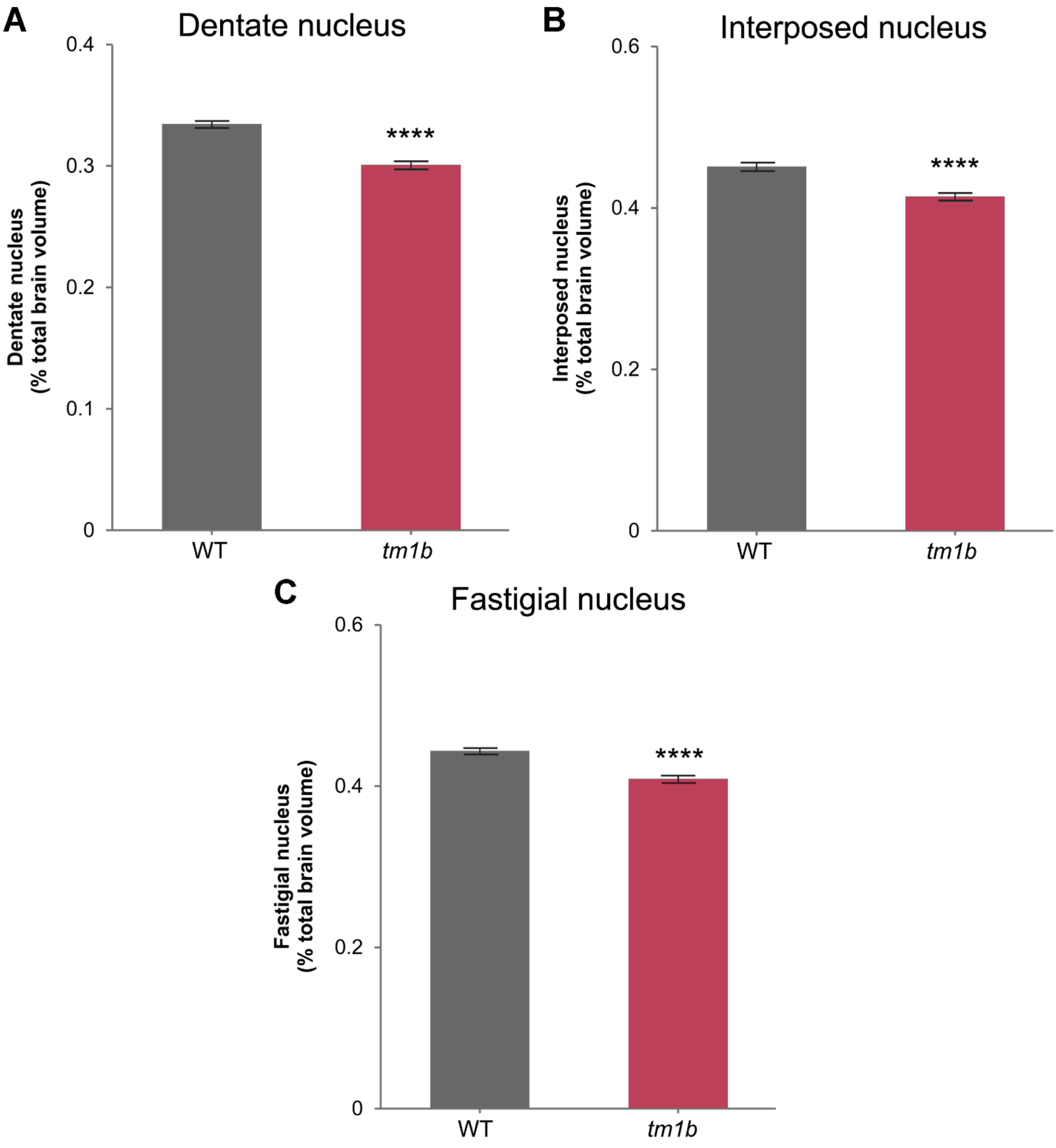


Relative (% total brain volume) volumetric differences in the dentate nucleus, interposed nucleus, and fastigial nucleus in *Pdzd8*$^{tm1b}$ mice (n = 32; 10♂, 22♀) and WT controls (n = 17; 7♂, 10♀) determined by high-resolution structural magnetic resonance imaging. **A** Dentate nucleus relative volume is decreased in *Pdzd8*$^{tm1b}$ mice by 10.06 ± 0.88% (unpaired $t$-test: $t = 8.45$, $p < 0.0001$). **B** Interposed nucleus relative volume is decreased in *Pdzd8*$^{tm1b}$ mice by 8.21 ± 0.80% (unpaired $t$-test: $t = 6.59$, $p < 0.0001$). **C** Fastigial nucleus relative volume is decreased in *Pdzd8*$^{tm1b}$ mice by 7.80 ± 0.85% (unpaired $t$-test: $t = 6.70$, $p < 0.0001$). *tm1b*, *Pdzd8*$^{tm1b}$ homozygous; WT, wild-type. ****$p < 0.0001$ versus WT.

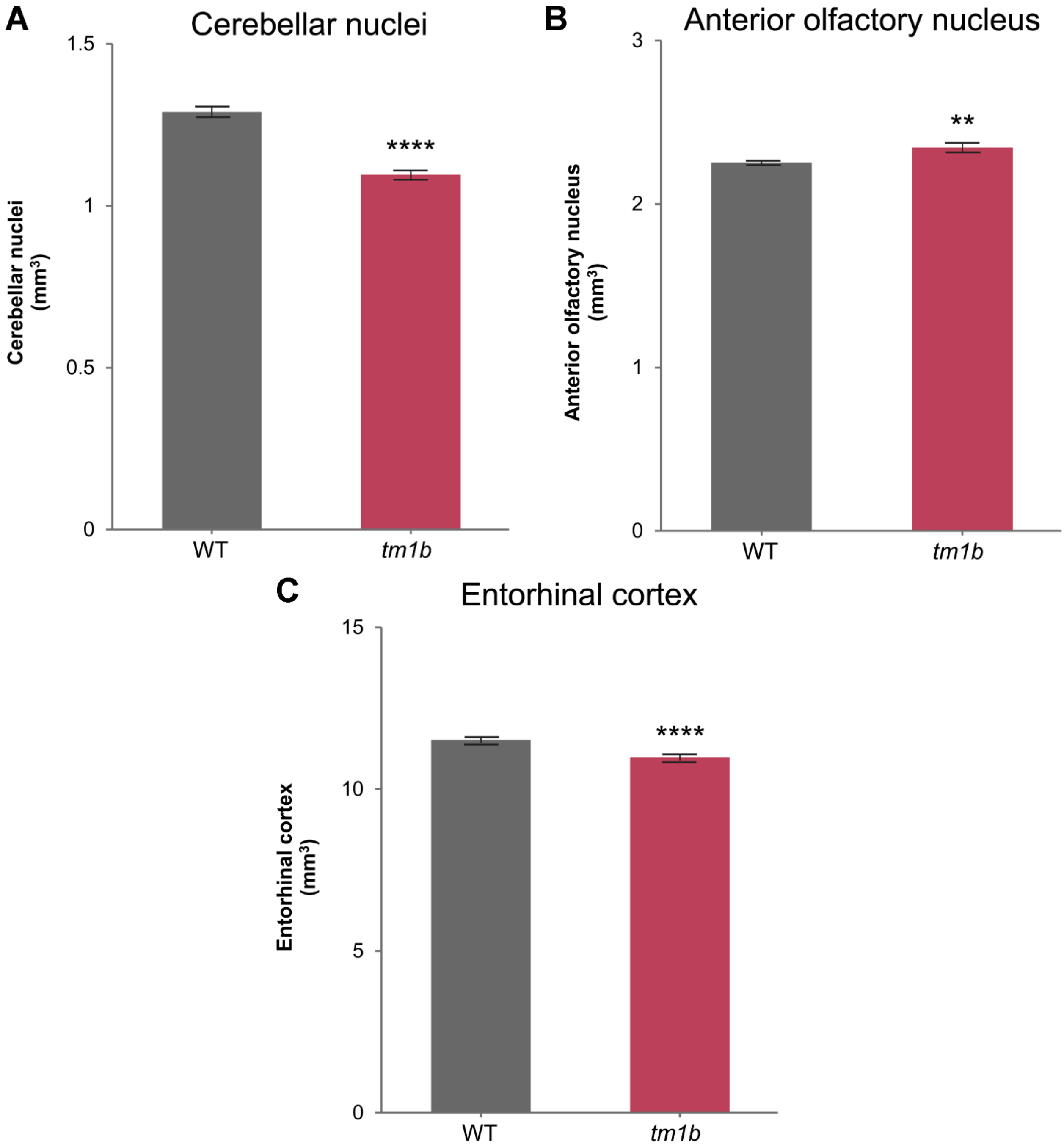


Absolute ($mm^3$) volumetric differences in the cerebellar nuclei, accessory olfactory bulb, and components of the primary olfactory cortex in *Pdzd8*$^{tm1b}$ mice (n = 32; 10♂, 22♀) and WT controls (n = 17; 7♂, 10♀) determined by high-resolution structural magnetic resonance imaging. **A** Cerebellar nuclei absolute volume is decreased in *Pdzd8*$^{tm1b}$ mice by 15.03 ± 1.04% (unpaired $t$-test: $t = 9.99$, $p < 0.0001$). **B** Anterior olfactory nucleus absolute volume is increased in *Pdzd8*$^{tm1b}$ mice by 4.04 ± 1.01% (unpaired $t$-test: $t = 3.65$, $p = 0.001$). **C** Entorhinal cortex absolute volume is decreased in *Pdzd8*$^{tm1b}$ mice by 4.73 ± 0.82% (unpaired $t$-test: $t = 4.16$, $p < 0.0001$). *tm1b*, *Pdzd8*$^{tm1b}$ homozygous; WT, wild-type. **$p < 0.01$, ****$p < 0.0001$ versus WT.
