# Supplementary material for: Autistic behavior is a common outcome of biallelic disruption of PDZD8 in humans and mice
Source: Mol Autism. 2025 Feb 27;16:14. doi: 10.1186/s13229-025-00650-8 (PMC11866840; doi:10.1186/s13229-025-00650-8)
Supplement: Supplementary file 5 — Supplementary Material 5 [file 13229_2025_650_MOESM5_ESM.pdf]

Additional File 5

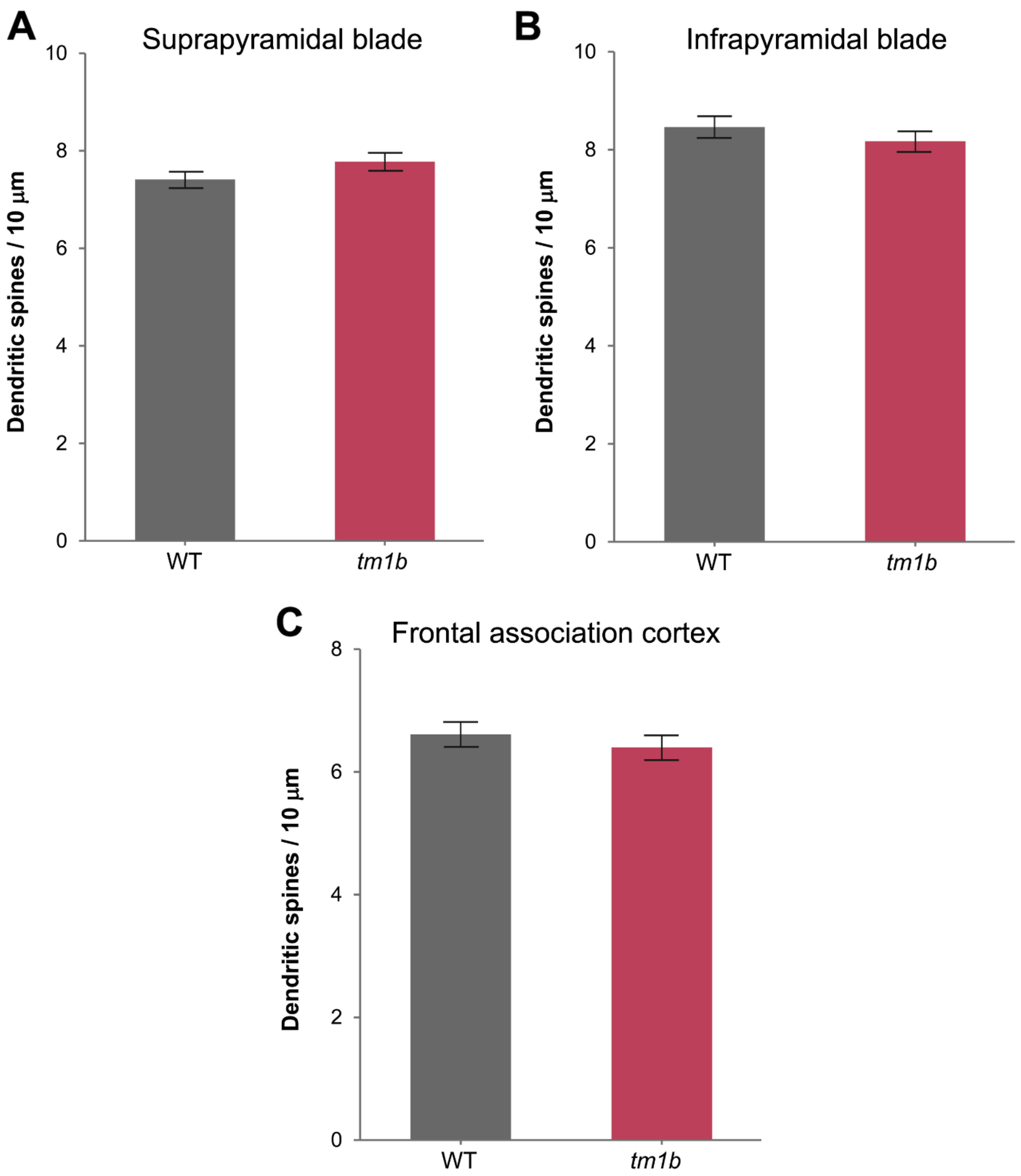

Unaltered density of dendritic spines in the suprapyramidal and infrapyramidal blades of the dentate gyrus and in the frontal association cortex in *Pdzd8*^{tm1b} mice. **A** Density of dendritic spines in the suprapyramidal blade of the dentate gyrus of *Pdzd8*^{tm1b} mice (n = 68 dendrites from n = 4♂ mice) and WT controls (n = 75 dendrites from n = 4♂ mice) (two-sample *t*-test: $t(141) = 1.58$, $p = 0.1163$). **B** Density of dendritic spines in the infrapyramidal blade of the dentate gyrus of *Pdzd8*^{tm1b} mice (n = 57 dendrites from n = 4♂ mice) and WT controls (n = 69 dendrites from n = 4♂ mice) (Mann–Whitney: $U = 1{,}870$, $p = 0.6302$). **C** Density of dendritic spines in the frontal association cortex of *Pdzd8*^{tm1b} mice (n = 70 dendrites from n = 4♂ mice) and WT controls (n = 80 dendrites from n = 4♂ mice) (two-sample *t*-test: $t(148) = 0.7655$, $p = 0.4452$). *tm1b*, *Pdzd8*^{tm1b} homozygous; WT, wild-type.
